# A mechanism-inspired UDP-*N*-acetylglucosamine pyrophosphorylase inhibitor†

**DOI:** 10.1039/c9cb00017h

**Published:** 2020-03-24

**Authors:** Olawale G. Raimi, Ramon Hurtado-Guerrero, Vladimir Borodkin, Andrew Ferenbach, Michael D. Urbaniak, Michael A. J. Ferguson, Daan M. F. van Aalten

**Affiliations:** Division of Gene Regulation and Expression, School of Life Sciences, University of Dundee Dow Street DD1 5EH Dundee UK dmfvanaalten@dundee.ac.uk; Wellcome Centre for Anti-Infectives Research, School of Life Sciences, University of Dundee Dow Street DD1 5EH Dundee UK

## Abstract

UDP-*N*-acetylglucosamine pyrophosphorylase (UAP1) catalyses the last step in eukaryotic biosynthesis of uridine diphosphate-*N*-acetylglucosamine (UDP-GlcNAc), converting UTP and GlcNAc-1P to the sugar nucleotide. Gene disruption studies have shown that this gene is essential in eukaryotes and a possible antifungal target, yet no inhibitors of fungal UAP1 have so far been reported. Here we describe the crystal structures of substrate/product complexes of UAP1 from *Aspergillus fumigatus* that together provide snapshots of catalysis. A structure with UDP-GlcNAc, pyrophosphate and Mg^2+^ provides the first Michaelis complex trapped for this class of enzyme, revealing the structural basis of the previously reported Mg^2+^ dependence and direct observation of pyrophosphorolysis. We also show that a highly conserved lysine mimics the role of a second metal observed in structures of bacterial orthologues. A mechanism-inspired UTP α,β-methylenebisphosphonate analogue (*me*UTP) was designed and synthesized and was shown to be a micromolar inhibitor of the enzyme. The mechanistic insights and inhibitor described here will facilitate future studies towards the discovery of small molecule inhibitors of this currently unexploited potential antifungal drug target.

## Abstract 2

UDP-*N*-acetylglucosamine pyrophosphorylase (UAP1) catalyses the last step in eukaryotic biosynthesis of uridine diphosphate-*N*-acetylglucosamine (UDP-GlcNAc), converting UTP and GlcNAc-1P to the sugar nucleotide.

## Graphical abstract

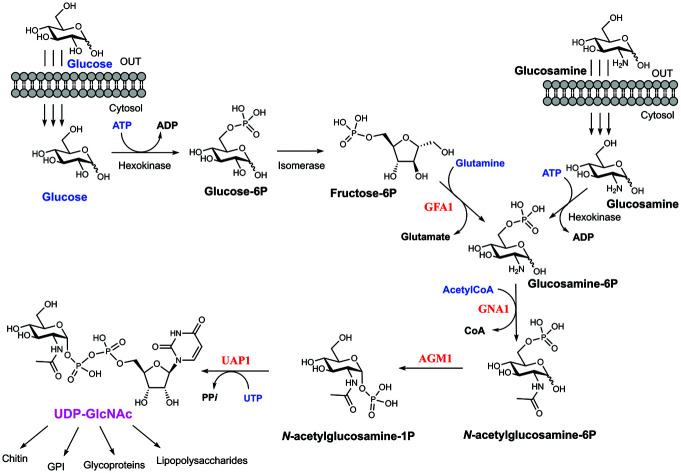

## Introduction

**Fig. 1:**
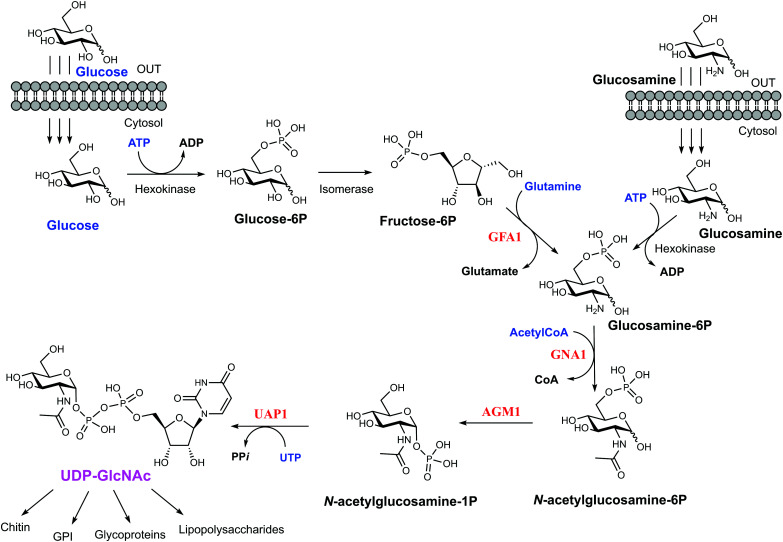
UDP-GlcNAc biosynthetic pathway. The four main enzymes of the pathway are coloured in red. Metabolites feeding the pathway are in blue and the final product of the pathway is coloured in pink. The various fates of UDP-GlcNAc are linked by the branching arrows.

Yeast and fungi are encapsulated by a cell wall that is important for morphogenesis and provides chemical/mechanical protection from the environment. Fungal cell walls are composed mainly of glucans, chitin and mannoproteins.^1^ Chitin is a β(1,4)-linked homopolymer of *N*-acetylglucosamine residues, accounting for only 1–2% of the cell mass in yeast,^2,3^ whereas the cell wall of filamentous fungi contains higher amounts of chitin, up to 10–20% of the dry weight.^4^ Chitin is synthesized from UDP-GlcNAc, which is also a sugar donor for the synthesis of glycoproteins, glycosaminoglycans and glycosylphosphatidylinositol (GPI) anchors in eukaryotes as well as molecules such as lipopolysaccharide in prokaryotes. In eukaryotes, UDP-GlcNAc is synthesized from fructose-6-phosphate (Fru-6P) and l-glutamine by a cascade of four enzymatic reactions (Fig. 1); (i) conversion of Fru-6P to GlcN-6P, (ii) acetylation of GlcN-6P to GlcNAc-6P, (iii) isomerization of GlcNAc-6P to GlcNAc-1P, and (iv) the uridylation of GlcNAc-1P to the sugar nucleotide UDP-GlcNAc. The enzymes involved in this pathway are (i) GlcN-6P synthase (GFA1), (ii) GlcN-6P acetyltransferase (GNA1), (iii) phosphoacetylglucosamine mutase (AGM1) and (iv) UDP-GlcNAc pyrophosphorylase (UAP1) (Fig. 1), respectively.^5^ Knock out studies of any of these genes have produced lethal phenotypes in a range of eukaryotes,^6–8^ with the exception of the knockout of GFA1, which produces GlcN/GlcNAc auxotrophic mutants,^9^ and the enzymes are considered to be potential drug targets. One species-specific allosteric inhibitor has been described for *T. brucei* UAP1,^10^ but no mechanism-based inhibitors have so far been reported for any of the last three enzymes in the pathway.

UDP-GlcNAc pyrophosphorylase (E.C.2.7.7.23) is a member of the large family of nucleotide diphosphate sugar pyrophosphorylases.^7^ Eukaryotic UDP-GlcNAc pyrophosphorylase catalyzes the synthesis of UDP-GlcNAc from UTP and GlcNAc-1P and also the reverse pyrophosphorolysis of UDP-GlcNAc (UTP + GlcNAc-1P ⇔ UDP-GlcNAc + PPi). Eukaryotic UAP1s have been categorized into mammalian UAP1 and non-mammalian UAP1.^11^ The crystal structures of the two isoforms (AGX1 and AGX2) of human UAP1 (hUAP1) were the first UAP1 structures to be reported.^12^ UAP1s consist of an N-terminal domain, a central pyrophosphorylase domain and a C-terminal domain. The functions of the N and C-terminal domains are not yet known.^11,12^ Non-mammalian UAP1s possess a primary structure similar to that of mammalian UAP1s, but lack an “insertion loop” of more than 10 amino acids residues^11^ as visualised in the crystal structures of *Candida albicans* and *Aspergillus fumigatus* UAP1s (*Ca*UAP1 and *Af*UAP1 respectively) (Fig. S1, ESI†).^11,13^ Together, these human and *C. albicans* UAP1 structures, solved in complex with substrates/products, suggest an S_N_2 reaction mechanism in which phosphate oxygen of GlcNAc-1P attacks the UTP α-phosphate, forming UDP-GlcNAc and releasing inorganic pyrophosphate (PPi). Although it has been shown that divalent cations facilitate the reactions catalysed by UAP1, their mechanistic roles are not clear, and the available structures have been determined in the absence of any such ions.

*Aspergillus fumigatus* is the major cause of Aspergillosis, an often invasive and potentially life-threatening infection, found most frequently in immunocompromised patients.^14,15^ As a step towards chemically validating *A. fumigatus* UDP-*N*-acetylglucosamine pyrophosphorylase (*Af*UAP1) as a potential drug target, we report the crystal structure of *Af*UAP1 in complex with the two reaction products, UDP-GlcNAc and pyrophosphate, and the cofactor magnesium ion, representing the first Michaelis complex for this enzyme family. Together with additional substrate/product complexes of this enzyme, this provides a range of snapshots of the reaction mechanism, revealing key conformational changes and essential participation of the magnesium ion and Lys437 in catalysis. A mechanism-inspired α,β-methylenebisphosphonate UTP analogue (*me*UTP) is the first example of a eukaryotic pyrophosphorylase inhibitor, providing a basis for the future design of small molecule inhibitor for this class of enzymes.

## Experimental

### Recombinant *Af*UAP1 production and structure determination

*Af*UAP1 was expressed as described recently.^13^ The protein (20 mg mL^−1^) was co-crystallized with UTP, GlcNAc-1P and UDP-GlcNAc respectively by incubating the protein with 5 mM of the ligands at 4 °C for 2 h before setting up crystal trays. Crystals grew after three days in a condition containing 0.2 M sodium formate and 20% PEG3350. To obtain the UAP1–UDP-GlcNAc–PPi–Mg^2+^ ion complex, an *Af*UAP1–UDP-GlcNAc crystal was soaked for 10 seconds in a cryo-protectant (mother liquor + 10% glycerol) containing 10 mM pyrophosphate and 10 mM magnesium. Prior to data collection, crystals were cryo-protected in mother liquor containing 10% glycerol. Data were collected at the European Synchrotron Radiation Facility (ESRF) (Grenoble, France) and processed with the HKL suite.^16^ The *Af*UAP1–UDP-GlcNAc structure and other complexes described here were solved by molecular replacement with MOLREP^17^ using the PDB entry 4BMA as the template.^13^ This gave a solution with two molecules in the asymmetric unit. The resulting electron density map was of good quality, allowing WarpNtrace^18^ to build a model containing most residues. REFMAC^19^ was used for further refinement, iterated with model building using COOT,^20^ producing a final models with the statistics shown in Table 1. Models for ligands were not included until their conformations were fully defined by unbiased |*F*_o_| − |*F*_c_|, *φ*_calc_ electron density maps. Ligand structures and topologies were generated by PRODRG.^21^ WHATIF^22^ was used to calculate hydrogen bonds and PyMOL was used to generate figures.^23^

**Table 1:** Diffraction data and refinement statistics

	UDP-GlcNAc + PPi, Mg^2+^	GlcNAc-1P	UTP
Resolution (Å)	20.0 (1.81–1.75)	20.0 (2.36–2.28)	20.0 (2.49–2.40)
Space group	*P*2_1_2_1_2_1_	*P*2_1_2_1_2_1_	*P*2_1_2_1_2_1_
Unit cell			
*a* (Å)	55.2	55.7	55.6
*b* (Å)	139.8	138.1	140.0
*c* (Å)	144.6	145.1	146.3
*α* = *β* = *γ* (°)	90	90	90
No. of reflections	441 409	157 074	177 172
No. of unique reflections	113 044	49 491	46 020
*I*/*σ* (*I*)	16.4 (2.4)	11.2 (2.4)	13.7 (3.2)
Completeness (%)	99.1	95.5	99.2
Redundancy	3.9 (3.8)	3.2 (3.0)	3.8 (3.0)
*R*_merge_ (%)	5.8 (56.8)	11.6 (39.2)	8.6 (32.5)
RMSD from ideal geometry			
Bond distance (Å)	0.008	0.009	0.010
Bond angle (°)	1.08	1.0	1.23
*R*_work_ (%)	21.0	20.6	22.7
*R*_free_ (%)	24.0	25.9	27.7
No. of residues	925	905	928
No. of water mol.	559	231	120
*B*-factors (Å^2^)			
Overall	23.8	48.6	34.8
Protein	23.4	48.8	34.8
Ligand	16.7	42.5	43.4
Solvent	29.3	44.5	29.2
PDB ID	6G9V	6TN3	6G9W

### Enzyme kinetics and inhibition

*Af*UAP1 steady-state kinetics was studied under linear conditions with not more than 10% of the substrate converted to products. The activity of the enzyme in the forward direction was determined as described previously.^13^ The enzymes were used at 20 nM with the exception of K148M mutant that was used at 185 nM. The absorbance intensity data were analyzed with nonlinear regression analysis using GRAFIT 5^24^ with the default equations for the first-order reaction rates and Michaelis–Menten steady-state kinetics. To investigate *Af*UAP1 inhibition by *me*UTP the forward reaction (formation of UDP-GlcNAc) was assayed using high performance anion exchange chromatography (HPAEC) based assay. The reaction mixtures (20 µM UTP, 50 µM GlcNAc-1P, 1 mM DTT, 5 nM enzyme, 50 mM Tris–HCl pH 8.0, 10 mM MgCl_2_ and 2% glycerol, total volume 100 µL) with and without varying concentrations of the inhibitor (0–1000 µM) were incubated for 30 min and the reactions were terminated by the addition of 10 µL of 0.1 M NaOH (∼10 mM final concentration) prior to analysis on a CarboPac-PA-1 column (Dionex) equilibrated with a 80 : 20 mixture of 1 mM NaOH and a 1 : 1 mixture of 1 mM NaOH and 1 M sodium acetate as described previously.^25^ The eluent was monitored at 260 nm and peaks were identified by comparison to standards. The IC_50_ was calculated using Graphpad Prism software, CA, USA (www.graphpad.com).

### Synthesis of an α,β-methylenebisphosphonate UTP (*me*UTP)

#### Compound **2** (Scheme 1)

To a hot (60 °C) solution of 2′,3′-*O*-isopropylidene uridine **1** (0.668 g, 2 mmol), triphenylphosphine (1.05 g, 4 mmol), and trimethyl methylenediphosphonate^26^ (0.88 g, 4 mmol) in THF (10 mL) diisopropylazodicarboxylate (1 mL, 5 mmol) was added dropwise. The reaction was further refluxed for 2 h, cooled to RT and concentrated. The residue was purified by flash chromatography in DCM–MeOH 0→10% to give 0.89 g (1.84 mmol, 92%) of the target product as a mixture of two isomers on the α-phosphorous atom.

HRMS (positive) *m*/*z* 501.1478; expected for C_17_H_31_N_2_O_11_P_2_ [M + H]^+^ 501.1403.

^1^H NMR (500 MHz, CDCl_3_) *δ* 10.20 (2 × d, 1H), 7.43 (d, *J* = 2.8, 0.5H), 7.41 (d, *J* = 2.7, 0.5H), 5.73 (d, *J* = 2.3, 0.5H), 5.69 (d, *J* = 2.3, 0.5H), 5.67 (d, *J* = 5.8, 0.5H), 5.65 (d, *J* = 5.8, 0.5H), 4.94–4.91 (m, 1H), 4.86 (dd, *J* = 6.4, 3.7, 0.5H), 4.82 (dd, *J* = 6.5, 3.5, 0.5H), 4.34–4.22 (m, 3H), 3.76–3.74 (m, 4.5H), 3.73–3.72 (m, 4.5H), 2.50 (dt, *J* = 21.2, 1.2, 2H), 1.49 (s, 3H), 1.27 (s, 3H).

^13^C NMR (126 MHz, CDCl_3_) *δ* 163.89, 150.36, 142.41, 142.33, 114.29, 102.49, 93.64, 93.30, 85.27, 85.22, 84.11, 80.50, 80.41, 77.50, 65.88, 65.82, 65.77, 53.42, 53.33, 53.27, 53.21, 52.44, 52.39, 26.97, 25.13, 23.66 (dt, *J* = 137.6, 8.2).

^31^P NMR (202 MHz, CDCl_3_) *δ* 21.81 (d, *J* = 6.4), 21.76 (d, *J* = 6.4), 21.70 (d, *J* = 6.4), 21.41 (d, *J* = 6.4).

#### Compound **3**

A solution of **2** (0.48 g, 1 mmol) in DCM (10 mL) and aqueous 90% trifluoroacetic acid (2 mL) was kept at RT for 1 h and concentrated. The residue was co-evaporated with toluene (2 × 2 mL). The final residue was purified by flash chromatography in DCM–MeOH 1→15% to give 0.379 g (0.85 mmol, 85%) of the intermediate diol. A solution of the above material (0.379 g, 0.85 mmol) in pyridine (5 mL) was treated with iso-butyric anhydride (0.415 mL, 2.5 mmol) in the presence of 4-(dimethylamino)pyridine (0.012 g, 0.1 mmol) at RT for 5 h. The reaction was quenched with MeOH (0.5 mL), kept for 10 min and concentrated. The residue was dissolved in DCM and washed successively with 1 M HCl, water, and a mixture of NaHCO_3_ saturated solution and brine. The organic layer was dried and concentrated. The residue was purified by flash chromatography DCM–MeOH 1→6% to give 0.43 g (0.74 mmol; 87%) of the target compound (two isomers on α-phosphorous atom).

HRMS (positive) *m*/*z* 858.1673; expected for C_21_H_35_N_2_O_13_P_2_ 585.1614 [M + H]^+^ 585.1614.

^1^H NMR (500 MHz, CDCl_3_) *δ* 10.10 (2 × d, 1H), 7.74 (d, *J* = 8.2, 0.5H), 7.67 (d, *J* = 8.2, 0.5H), 6.13 (dd, *J* = 6.6, 1.3, 0.5H), 5.77 (dd, *J* = 8.1, 1.9, 0.5H), 5.73 (dd, *J* = 8.1, 1.9, 0.5H), 5.44 (dd, *J* = 5.9, 3.7, 0.5H), 5.37 (dd, *J* = 5.9, 3.5, 0.5H), 5.30 (t, *J* = 6.2, 0.5H), 5.25 (t, *J* = 6.4, 0.5H), 4.45–4.41 (m, 0.5H), 4.39–4.32 (m, 1H), 4.24–4.20 (m, 1H), 3.82–3.74 (m, 9H), 2.62–2.46 (m, 4H), 1.14 (bs, 3H), 1.13 (bs, 3H), 1.10–1.07 (m, 6H).

^13^C NMR (126 MHz, CDCl_3_) *δ* 175.79, 175.69, 175.51, 163.39, 163.36, 150.69, 150.65, 140.07, 139.99, 103.50, 103.36, 86.13, 85.89, 81.21, 81.15, 81.04, 80.98, 77.37, 72.38, 72.31, 69.91, 69.87, 65.37, 65.34, 53.33 (m), 33.65, 33.54, 23.93 (dt, *J* = 137.3, 14.2) 18.82, 18.71, 18.63.

^31^P NMR (202 MHz, CDCl_3_) *δ* 22.44 (d, *J* = 7.3), 21.87 (d, *J* = 6.6), 21.46 (d, *J* = 7.3), 21.41 (d, *J* = 6.7).

#### Compound **4**

To a solution of **3** (0.06 g, 0.1 mmol) in acetonitrile (1 mL) bromotrimethylsilane (0.13 mL, 1 mmol) was added at RT and the reaction was kept for 2 h. The reaction was concentrated and the residue was co-evaporated with aqueous MeOH (10 : 1) (1 mL). The residue was co-evaporated with toluene (2 × 1 mL) to remove traces of water. The residue was dissolved in anhydrous MeOH (1 mL) and treated with a 25% stock solution of NaOMe (0.05 mL) at room temperature (RT) for 3 h. The reaction was quenched with Dowex50W8-Et_3_NH^+^. The resin was filtered off and the filtrate was concentrated. The residue was dissolved in water and freeze-dried to give 0.072 g (0.1 mmol) of the target compound as a fluffy white solid.

HRMS (negative) *m*/*z* 401.0149; expected for C_10_H_15_N_2_O_11_P_2_ [M − H]^−^ 401.0151.

^1^H NMR (500 MHz, MeOD) *δ* 7.76 (d, *J* = 8.1, 1H), 5.73 (d, *J* = 4.6, 1H), 5.71 (d, *J* = 8.1, 1H), 4.14 (dt, *J* = 9.8, 5.2, 2H), 4.05–4.00 (m, 1H), 3.98–3.87 (m, 2H), 2.96 (q, *J* = 7.3, 12H), 1.97 (dd, *J* = 27.1, 11.5, 2H), 1.03 (t, *J* = 7.3, 18H).

^13^C NMR (126 MHz, MeOD) *δ* 166.20, 151.74, 141.81, 102.46, 88.37, 83.31 (d, *J* = 7.6), 73.69, 69.54, 63.28 (d, *J* = 4.3), 46.57, 27.19 (t, *J* = 125.0), 8.

^31^P NMR (202 MHz, MeOD) *δ* 16.43, 14.2.

#### Compound **5**

To a solution of **4** (0.072 g, 0.1 mmol) in DMF (1.5 mL) was added *N*,*N*′-carbonyldiimidazole (0.057 g, 0.35 mmol) and the mixture was stirred for 3 h at RT. The reaction was quenched by addition of MeOH (0.01 mL) and concentrated. The residue was co-evaporated with DMF (2 × 1 mL) and dissolved in a fresh portion of DMF (1 mL). A 0.5 M solution of tributylammonium phosphate in DMF (1 mL) was added to the above solution at RT, which resulted in immediate formation of white precipitate. The reaction was further stirred at RT for 16 h. The reaction was diluted with water (2 mL) and concentrated. The residue was dissolved in water and purified by ion-exchange chromatography using Fast Flow Sepharose Q (15 × 200 column); linear gradient NH_4_HCO_3_ 0.04 to 0.4 M, flow-rate 5 mL min^−1^; detection on 214 and 254 nm. The appropriate fractions were pooled and freeze-dried to give 0.0085 g (0.015 mmol, 15%) of the target compound as white fluffy substance.

HRMS (negative) *m*/*z* 480.9857; expected for C_10_H_16_N_2_O_14_P_3_ [M − H]^−^ 480.9814.

^1^H NMR (500 MHz, D_2_O) *δ* 7.89 (d, *J* = 8.1, 1H), 5.85–5.81 (m, 2H), 4.29–4.25 (m, 2H), 4.13 (d, *J* = 1.9, 1H), 4.10–4.01 (m, 2H), 2.29–2.18 (dt, *J* = 21, 2, 2H).

^31^P NMR (202 MHz, D_2_O) *δ* 17.80 (d, *J* = 9.3), 6.18 (dd, *J* = 23.6, 9.3), −9.25 (d, *J* = 23.6).

## Results and discussion

### *Af*UAP1 undergoes large conformational changes during catalysis

**Fig. 2:**
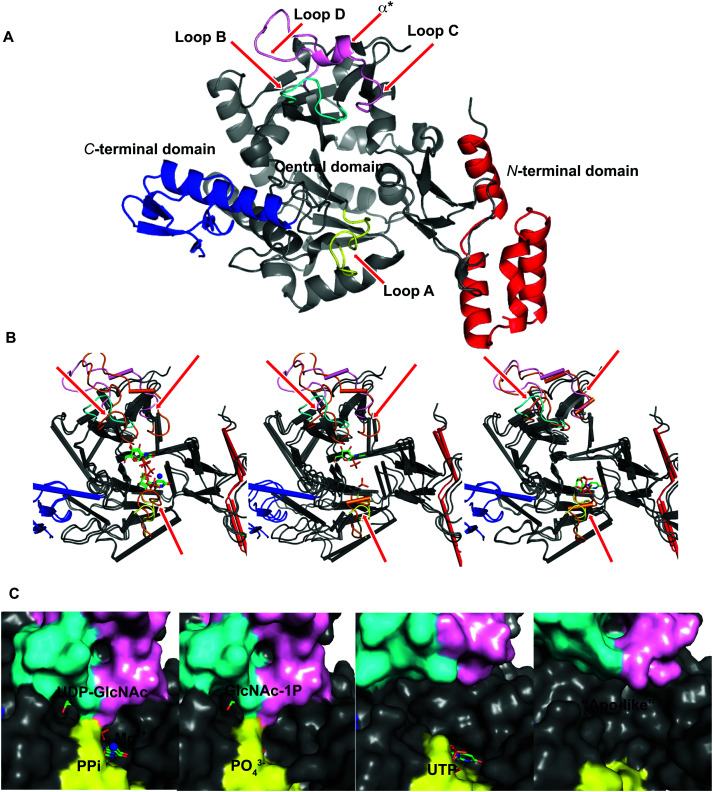
Ligand-induced conformational changes of *Af*UAP1. (A) Cartoon representation of *Af*UAP1, the N-terminal domain is coloured red, the C-terminal domain is coloured blue and the pyrophosphorylase domain is coloured in grey. Loops A, B, and C–D together with α* are coloured in yellow, cyan and pink, respectively. The flexible regions are indicated by red arrows. (B) Cartoon representation of the superposed “apo-like” structure with the different complexes showing a large area around the active site. While the colours for the mobile regions are maintained for the “apo-like” structure, the same loops and α* are coloured in orange in the different complexes. (C) The surface representation shows conformational changes around the active site in the various *Af*UAP1 complexes. The substrates and the products are shown as sticks, the Mg^2+^ ion is shown as a blue sphere.

To explore UAP1 catalytic mechanism and provide a structural framework for the design and optimization of inhibitors, crystal structures of *Af*UAP1 in complex with substrates/products were determined. Initially, a high-resolution dataset of a Michaelis complex of the enzyme at 1.75 Å was trapped using crystals grown in the presence of UDP-GlcNAc and subsequently soaked with PPi and Mg^2+^ (Table 1 and Fig. 2; RMSD of 1.6 Å on Cα atoms compared to the “apo-like” structure described earlier with PDB 4BMA^13^). One of the two protein molecules in the asymmetric unit showed an empty active site, and is considered here as the “apo-like” form (Fig. 2). To understand the catalytic mechanism, two further complexes were also determined – a substrate complex with GlcNAc-1P and a substrate complex with UTP (Table 1 and Fig. 2).

**Fig. 3:**
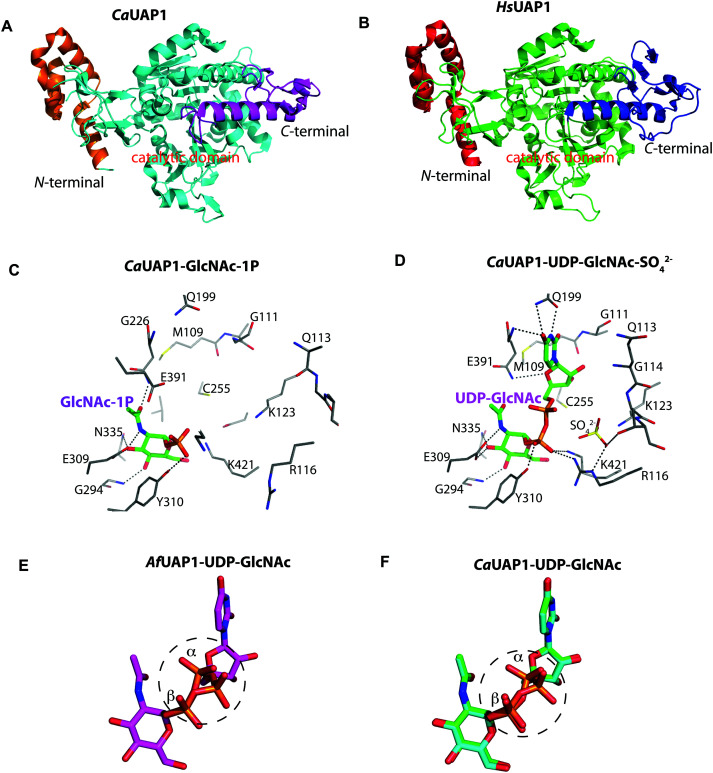
Comparison of *Af*UAP1 with other UAP1s. (A) Overall structure of *Ca*UAP1. Coloured in orange, cyan and purple are the N-terminal, catalytic and C-terminal domains, respectively. (B) Overall structure of *Hs*UAP1. Coloured in red, green and blue are the N-terminal, catalytic and C-terminal domains, respectively. (C) Active site of *Ca*UAP1 with GlcNAc-1P bound. Active site residues are shown in sticks. Hydrogen bond interactions are shown as black dashes. (D) Active site of *Ca*UAP1 with UDP-GlcNAc–SO_4_^2−^ bound. Active site residues are shown in sticks. Hydrogen bond interactions are shown as black dashes. SO_4_^2−^ is shown as sticks. (E) Stick representation of UDP-GlcNAc in the *Af*UAP1–UDP-GlcNAc–PPi–Mg^2+^ ternary complex, showing the observed dual conformation around the α-phosphate. (F) Superposition of UDP-GlcNAc from the *Ca*UAP1–UDP-GlcNAc product complex (PDB: 2yqs, blue) with UDP-GlcNAc from the *Ca*UAP1–UDP-GlcNAc reaction-completed product complex (PDB: 2yqj, green). This superposition recapitulates the dual conformation observed in the *Af*UAP1–UDP-GlcNAc–PPi–Mg^2+^ complex.

**Fig. 4:**
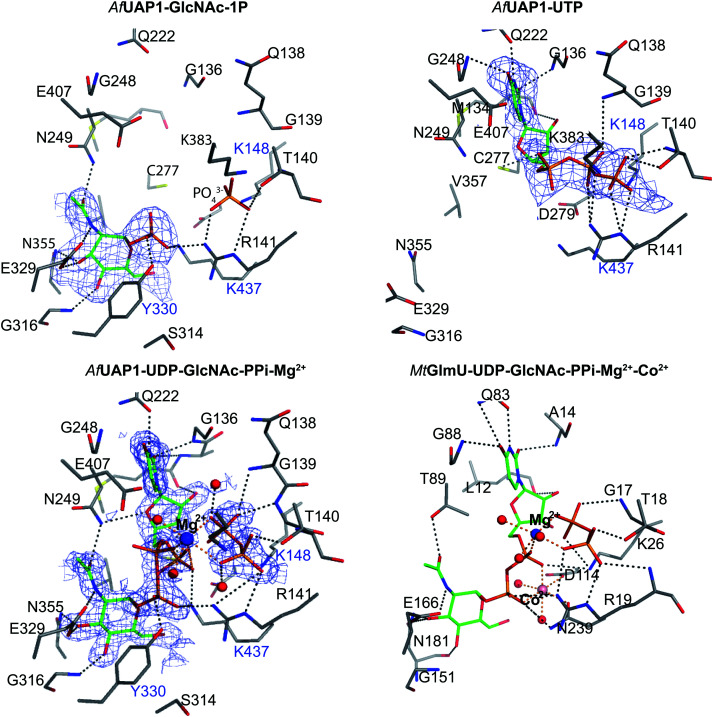
Active site of *Af*UAP1 complexes and comparison with the *Mt*GlmU ternary complex. Amino acids and ligands are shown as sticks with grey and green carbon atoms respectively. Water molecules, Mg^2+^ and Co^2+^ atoms are shown as spheres in red, blue and pink respectively. Hydrogen bonds and metal coordination are represented with dotted lines coloured black and orange respectively. The unbiased |*F*_o_| − |*F*_c_|*φ*_calc_ electron density maps for the ligands is shown in blue, contoured from 2.2 to 2.5*σ* depending on the ligand. The blue characters indicate the residues mutated in the mutagenesis assay.

The overall structure of *Af*UAP1 is similar to that previously described for eukaryotic UAP1s *Ca*UAP1^11^ and *Hs*UAP1^12^ (Fig. 3A and B). *Af*UAP1 and *Ca*UAP1 share 52% sequence identity (Fig. S1, ESI†) and superposition of the overall structures of these two proteins reveals an RMSD of 1.1 Å (on all Cα atoms), indicating a similar overall structure. *Af*UAP1 possesses a three-domain fold with the central catalytic domain flanked by two domains of unknown function (Fig. 2A). While the N-terminal domain of *Af*UAP1 consists entirely of four α-helices (Fig. 2A), the central domain consists of seventeen β-strands and twelve helices adopting the Rossmann fold. The C-terminal domain consists of four short β-strands and a long helix that connects to the central domain (Fig. 2A). *Af*UAP1 has a large active site cleft, with its wall being formed by the central domain and the C-terminal domains (Fig. 2). Ligand binding at the bottom of the active site induces a number of significant conformational changes compared to the “apo-like” structure. The binding of GlcNAc-1P to *Af*UAP1 is stabilized by hydrogen bonds with Asn249, Gly316, Tyr330, Glu329, Asn355 and Lys437. Interestingly, a PO_4_^3−^ molecule which occupies the same position as the UTP γ-phosphate interacts with R141 and T140 (Fig. 2B, C and 4) and induce a shift of loops A, B, C, D and α* on the central domain, closing the active site (Fig. 2A and B) (RMSD *vs.* “apo-like” structure = 2.1 Å on all Cα atoms). Similar interactions were seen in the *Ca*UAP1–GlcNAc-1P complex with equivalent residues (Tyr310, Asn227, Glu309, Gly294, Asn335, and Lys421) except for the absence of the PO_4_^3−^ molecule, which prevents the ordering of R116 (R141 in *Af*UAP1) (Fig. 3C and D).^11^ We hypothesise that in the absence of this PO_4_^3−^ molecule, the binding of GlcNAc-1P alone would only cause partial closure of the active site. In contrast, UTP appears to bind *Af*UAP1 with no significant conformational changes (RMSD *vs.* “apo-like” structure = 1.1 Å on all Cα atoms), interacting with Gly136, Gln138, Gly139, Thr140, Lys148, Arg141, Gln222, Gly248, Cys277 and Lys383 (Fig. 2B, C and 4). However, binding of UDP-GlcNAc, magnesium and PPi to the enzyme causes a considerable change to the overall structure of the protein (Fig. 2B, C and 3) (RMSD *vs.* “apo-like” structure = 2.1 Å on all Cα atoms). UDP-GlcNAc binds to the enzyme with the GlcNAc and uridine moieties of the molecule occupying positions equivalent to GlcNAc-1P (max. atomic positional shift = 0.6 Å) and UTP (max. atomic positional shift = 1.0 Å), also establishing similar hydrogen bonding interactions (Fig. 2B, C and 4). Further hydrogen bonds are established between α and β phosphates of UDP-GlcNAc and Arg141 and Lys437 (Fig. 4). The PPi molecule occupies a position approximately equivalent to the β and γ-phosphates of UTP (max. positional shift on phosphorus atoms = 1.5 Å), and is tethered by interactions with positively charged residues, such as Arg141, Lys437 and Lys148 (Fig. 4). A comparison of *Af*UAP1–UDP-GlcNAc–Mg^+2^–PPi and *Ca*UAP1–UDP-GlcNAc complexes reveals that UDP-GlcNAc occupies the same position and makes identical interactions with equivalent residues in both enzymes (Fig. 3C and D).^11^ Interestingly a SO_4_^2−^ ion occupies the same position as one of the phosphates of the PPi seen in the *Af*UAP1–UDP-GlcNAc–PPi–Mg^2+^ complex (Fig. 3D and 4). Taken together, it appears that to accommodate substrates and release products, *Af*UAP1 undergoes large conformational changes during catalysis.

### The Mg^2+^ ion aligns the reactants for catalysis

**Fig. 5:**
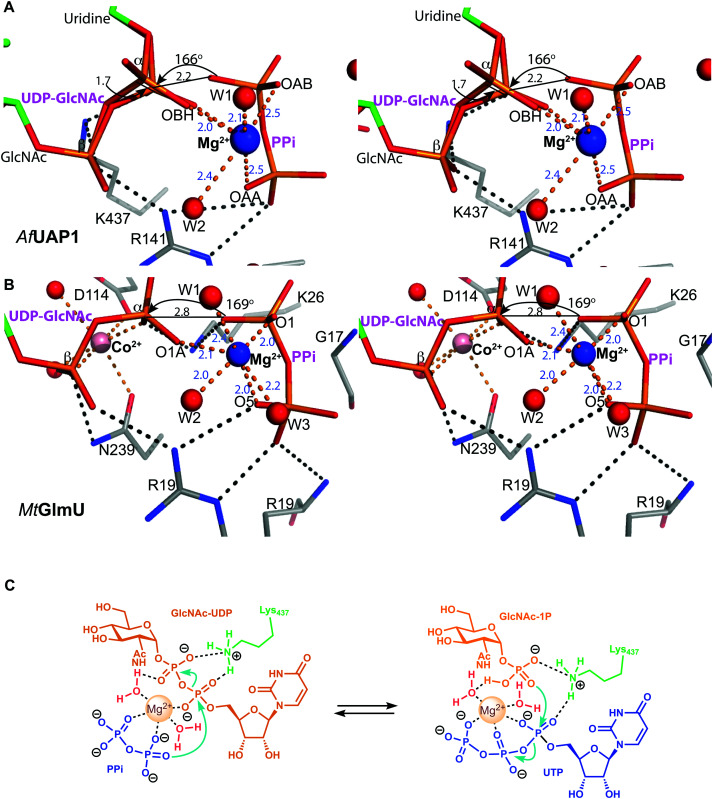
Magnesium coordination and *Af*UAP1 mechanism. (A) Magnesium coordination in the *Af*UAP1–UDP-GlcNAc–PPi–Mg^2+^ complex active site. A view of the active site of the *Af*UAP1–UDP-GlcNAc–PPi–Mg^2+^ and *Mt*GlmU–UDP-GlcNAc–PPi–Mg^2+^–Co^2+^ complexes. The attack distance for pyrophosphorolysis is 2.2 and 2.8 Å and the attack angle is 166° and 169° for UAP1 and GlmU, respectively. The distance between the α-phosphates in both UDP-GlcNAc adopting the dual conformation is 1.7 Å. The Mg^2+^ is tetragonal bipyramidally coordinated (albeit with a disordered 6th ligand in *Af*UAP1) as suggested by coordination distances and angles. (B) Magnesium coordination in the *Mt*GlmU–UDP-GlcNAc–PPi–Mg^2+^–Co^2+^ complex active site. (C) Schematic diagram of the proposed forward and reverse reaction mechanisms. The left panel indicates chemical bond formation/cleavage (cyan arrows) in the *Af*UAP1–PPi–UDP-GlcNAc ternary complex shown in Fig. 5A leading to the formation of GlcNAc-1P and UTP. The right panel using the same ternary complex structure as a template indicates chemical bond formation/cleavage in the reverse reaction leading to the formation of UDP-GlcNAc and PPi from GlcNAc-1P and UTP is proposed.

Several reports have shown that UAP1 requires divalent cations, such as Mg^2+^, for full activity.^7,11,12^ The UDP-GlcNAc–PPi–Mg^2+^ Michaelis complex of the reverse reaction trapped here reveals the key role the metal plays in coordinating the reactants/products (Fig. 4 and 5). The magnesium ion with an occupancy of 1.0, is pentacoordinated by the phosphate groups of pyrophosphate, UDP-GlcNAc and two water molecules in a tetragonal bipyramidal arrangement (distances ranging between 2.0 and 2.4 Å) while the 6th ligand is absent (presumably a disordered water molecule), providing the necessary geometry to place the attacking oxygen of pyrophosphate within 2.2 Å distance (angle of attack 166°) from the UDP-GlcNAc α-phosphate (Fig. 5A), compatible with a direct displacement mechanism (Fig. 4). Interestingly, the high-resolution electron density map also reveals an alternative conformation for the UDP-GlcNAc α-phosphate that flips 1.7 Å resulting in an “off conformation”, and placed 3.5 Å from the PPi oxyanion (Fig. 4 and 5). It is possible that this “off conformation” resembles the product of the forward synthesis preceding a conformation that is the Michaelis complex of the reverse pyrophosphorolysis reaction. Interestingly, superposition of two separate *Ca*UAP1–UDP-GlcNAc complexes (PDB entries 2yqs and 2yqj^11^) reveals a similar dual conformation as observed in the *Af*UAP1–UDP-GlcNAc–PPi–Mg^2+^ complex described here (Fig. 3E and F). The role of the metal is firstly to correctly align the UTP with GlcNAc-1P, and UDP-GlcNAc with PPi, and secondly, to facilitate close proximity of the negatively charged phosphate groups. Similarly, Pelissier *et al.*^27^ also proposed that Mg^2+^ prevents electrostatic repulsion between phosphate groups of the substrates Man-1P and GTP of the thermophilic bacterium *Thermotoga maritima* pyrophosphorylase. Thus, the Mg^2+^ ion aligns the reactants for catalysis.

### A conserved lysine occupies the position of a second metal ion in GlmU

The structure suggests that the reverse (pyrophosphorolysis) reaction may be facilitated by an increase in electrophilicity of the α-phosphorus induced by a salt bridge interaction between a lysine that is conserved in eukaryotes (Lys437 in *Af*UAP1, Fig. S1, ESI†) and the α phosphate of UDP-GlcNAc (Fig. 5). Similarly, Lys437 may facilitate the forward reaction by inducing an increase of the electrophilic character of the UTP α-phosphorus (Fig. 5).

Recently the structures of a ternary complex of a bacterial UAP from *M. tuberculosis* named *Mt*GlmU, and other classes of nucleotidyltransferases,^28,29^ revealed an unusual catalytic mechanism dependent on two-metal-ions^30^ (Fig. 4 and 5B). GlmUs are bacterial enzymes with two domains catalysing two independent reactions. While the C-terminal domain catalyses an acetyltransfer reaction from acetyl-CoA to glucosamine-1P (GlcN-1P) to form *N*-acetylglucosamine-1P (GlcNAc-1P), the N-terminal domain possesses uridylation activity converting GlcNAc-1P to UDP-GlcNAc in the presence of UTP.^30^ In the *Mt*GlmU structure, UDP-GlcNAc and PPi interact through hydrogen bonds with Ala14, Gly17, Thr18, Gln83, Gly88, Thr89, Gly151, Glu166 and Asn181, and salt bridges with Lys26 and Arg19 (Fig. 4). The magnesium atom in both the *Af*UAP1 and *Mt*GlmU ternary complexes occupy similar positions but they differ in how they are coordinated (Fig. 5). The metal ion in *Af*UAP1 is pentacoordinated, whereas the magnesium atom in GlmU is hexacoordinated by the phosphate groups of PPi and UDP-GlcNAc, and three water molecules (Fig. 5). The average bond angle of coordination of magnesium in the *Af*UAP1–UDP-GlcNAc–PPi–Mg^2+^ complex is 89 ± 10° while in the *Mt*GlmU–UDP-GlcNAc–PPi–Mg^2+^–Co^2+^ complex it is 90 ± 17°. A comparison of the angles of magnesium coordination in *Af*UAP1 and *Mt*GlmU complexes is shown in Table 2. With respect to the reaction mechanism, the pyrophosphate is within 2.8 Å (with an angle of attack of 169°) from the UDP-GlcNAc α-phosphate and therefore at a distance not as optimal as that found in the Michaelis complex of *Af*UAP1, suggesting that a positional shift of UDP-GlcNAc α-phosphate might be required in order to facilitate catalysis (Fig. 5A).

**Table 2:** Measurements of angles between Mg^2+^ and ligands in the *Af*UAP1–UDP-GlcNAc–PPi–Mg^2+^ and *Mt*Glmu–UDP-GlcNAc–PPi–Mg^2+^–Co^2+^ complexes

*Af*UAP1–UDP-GlcNAc–PPi–Mg^2+^		*Mt*Glmu–UDP-GlcNAc–PPi–Mg^2+^–Co^2+^	
Atoms	Angle (°)	Atoms	Angle (°)
W1Mg^2+^W2	100.1	W1Mg^2+^W2	80.7
W1Mg^2+^W3	nd	W1Mg^2+^W3	103.3
W1Mg^2+^OBH	87.9	W1Mg^2+^O1A	88.7
W1Mg^2+^OAB	105.2	W1Mg^2+^O1	107.9
W3Mg^2+^OAA	nd	W3Mg^2+^O5	69.6
W3Mg^2+^W2	nd	W3Mg^2+^W2	69.7
OAAMg^2+^OAB	79.7	O5Mg^2+^O1	80.0
OABMg^2+^OBH	82.8	O1Mg^2+^O1A	93.9
W3Mg^2+^OAB	nd	W3Mg^2+^O1	116.1
W2Mg^2+^OAA	76.0	W2Mg^2+^O5	92.1
W2Mg^2+^OBH	85.0	W2Mg^2+^O1A	76.4
OAAMg^2+^OBH	96.1	O1AMg^2+^O5	94.2

Interestingly, the crystal structure reveals the presence of a second metal in the *Mt*GlmU identified as cobalt, although experiments in solution confirm that GlmU requires two magnesium ions for catalysis.^30^ Furthermore, this second metal binding site is found in a position occupied by Lys437 in *Af*UAP1 (Fig. 4 and 5B). In the *C. albicans* UAP1 crystal structure, Lys421 occupies a similar position and was also predicted to play the role of the second metal ion.^11^ In *Leishmania major* UDP–glucose pyrophosphorylase^31^ and a bacterial GDP–mannose pyrophosphorylase,^27^ a lysine was also observed to occupy the position of the second Mg^2+^ seen in *C. glutamicum* UGP.^29^ It is possible that bacterial GlmUs and eukaryotic UAP1s follow a similar catalytic mechanism but differ in the number of metal atoms and consequently in how the electrophilic character of α-phosphorous is enhanced. In eukaryotes a single magnesium atom is essential for the reaction while the position of the second magnesium atom present in bacteria is occupied by a basic amino acid. Thus, a conserved lysine in *Af*UAP1 occupies the position of a second metal ion in GlmU.

### Lys437 is required for *Af*UAP1 catalysis

Several of the amino acid residues (*e.g.* equivalents of Thr140, Arg141 and Lys148 in *Af*UAP1, Fig. 4) shown to bind the phosphates of nucleotides/phosphosugars in complexes with other UAP1 enzymes have been mutated previously and shown to be important for substrate binding/catalysis.^7^ However, to probe the role of Lys437 in catalysis, we carried out site-directed mutagenesis of this basic residue and two other amino acids involved in stabilization of substrates/products.^7,11,12^ The mutants have the same expression levels as the wild type enzyme as confirmed by SDS-PAGE (Fig. S2A, ESI†). As mentioned earlier, Lys437 makes direct contacts with the phosphate group of GlcNAc-1P (Fig. 4) and with the α and β-phosphate of UDP-GlcNAc (Fig. 4). Tyr330 establishes a hydrogen bond with the phosphate group of GlcNAc-1P, and Lys148 is involved in UTP binding, contacting the phosphate groups of UTP and pyrophosphate (Fig. 4) through direct and indirect hydrogen bonds. In agreement with the structural data, the K148M and Y330F mutants affect the *K*_m_ values of UTP (10-fold increase) and GlcNAc-1P (6-fold increase), respectively (Table 3). The K437A mutant shows an increase in the *K*_m_ values of both substrates (32 and 10-fold increase in the *K*_m_s of GlcNAc-1P and UTP respectively), which may be the result of disrupting the interaction of Lys437 with the phosphate groups of GlcNAc-1P and UDP-GlcNAc (Fig. 4). To probe the effect of this residue further we made K437R and K437M mutants. The K437R mutant shows a slight increase in *K*_m_ by 5- and 4-fold for GlcNAc-1P and UTP, respectively while the K437M mutant completely abolishes enzyme activity (Table 3 and Fig. S2B, ESI†). The increase in activity by the K437R mutant compared to the K437A could be as a result of the arginine mimicking the positive charge present on Lys437, which is essential for an optimal catalytic efficiency. However, the activity of K437R is 2.9-fold lower than the wild type enzyme likely due to the bulkier arginine side chain not being positioned optimally with respect to the α-phosphate group of either UTP or UDP-GlcNAc. Overall, all the four mutants reduce *k*_cat_ (Table 3). Out of the five mutants tested, K437A has the largest effect on catalytic efficiencies with a 433 and 117-fold reduction towards GlcNAc-1P and UTP, respectively (Table 3) and K437M completely inactivates the enzyme. Therefore, our data suggest that these residues play key roles in aligning the substrates for UAP1-catalyzed UDP-GlcNAc synthesis/pyrophosphorolysis, in particular supporting the role of Lys437 as being essential for catalysis in line with the proposed catalytic mechanism (Fig. 5C).

**Table 3:** Kinetic parameters of the forward reaction for the wild type and mutant forms of *Af*UAP1. The results are the mean ± SD for three determinations

	GlcNAc-1P	UTP			GlcNAc-1P	UTP
	*K*_m_ (µM)	*K*_m_ (µM)	*V*_max_ (µM s^−1^)	*k*_cat_ (s^−1^)	*k*_cat_/*K*_m_ (s^−1^ µM^−1^)	*k*_cat_/*K*_m_ (s^−1^ µM^−1^)
WT	34 ± 3	21 ± 2	0.044 ± 0.001	2.2 ± 0.3	0.065	0.10
K148M	44 ± 13	200 ± 31	0.0074 ± 0.0007	0.4 ± 0.01	0.0091	0.002
Y330F	200 ± 72	25 ± 5	0.016 ± 0.001	0.8 ± 0.1	0.004	0.032
K437A	1100 ± 900	200 ± 62	0.0033 ± 0.0004	0.17 ± 0.02	0.00015	0.00085
K437R	155 ± 29	88 ± 12	0.027 ± 0.003	1.3 ± 0.1	0.008	0.015
K437M	nd	nd	nd	nd	nd	nd

### Design and synthesis of *me*UTP as a mechanism-inspired inhibitor

**Fig. 6:**
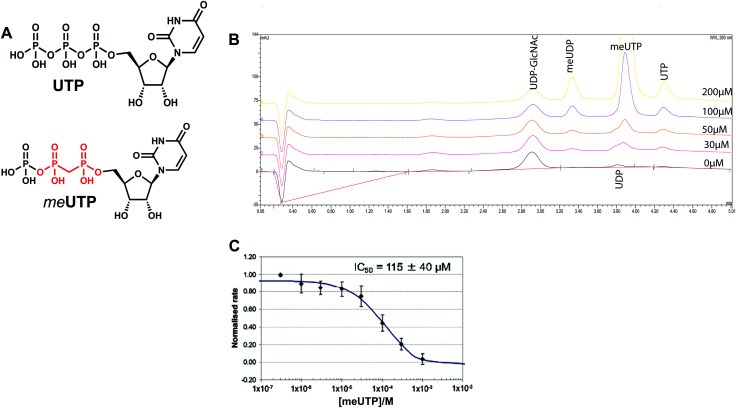
Kinetic characterization of *me*UTP (compound **5**). (A) Chemical structures of UTP and *me*UTP. The α,β-methylenebisphosphanate is indicated in red. (B) HPAEC chromatogram of *Af*UAP1 inhibition by *me*UTP, where [UTP] ≈ *K*_m_ and [GlcNAc-1-P] is in excess. The inhibitor concentration is indicated on each curve (0–200 µM). The reaction mixtures (20 µM UTP, 50 µM GlcNAc-1P, 1 mM DTT, 5 nM enzyme, 50 mM Tris–HCl pH 8.0, 10 mM MgCl_2_ and 2% glycerol, total volume 100 µL) with and without varying concentrations of the inhibitor were incubated for 30 min and the reactions were terminated by the addition of 10 µL of 0.1 M NaOH (∼10 mM final concentration) prior to analysis on a CarboPac-PA-1 column (Dionex) equilibrated with a 80 : 20 mixture of 1 mM NaOH and a 1 : 1 mixture of 1 mM NaOH and 1 M sodium acetate. The eluent was monitored at 260 nm and peaks were identified by comparison to standards. (C) IC_50_ curves of *me*UTP with fixed [UTP] and [GlcNAc-1P] and varying concentration of inhibitor.

**Scheme 1:**
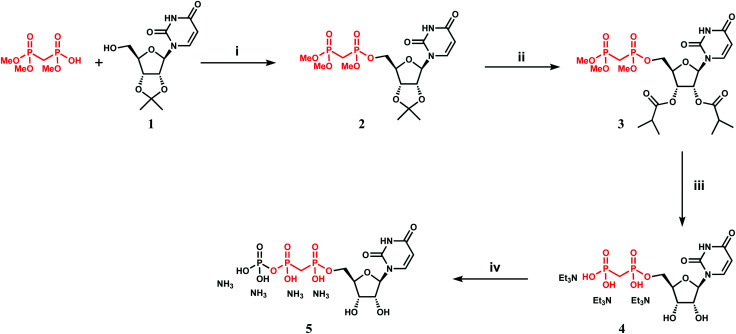
Synthesis of α,β-methylenebisphosphanate UTP analogue (*me*UTP). *Reagents and conditions*: (i) DIAD, PPh_3_, trimethyl methylenediphosphonate, THF, 2 h, reflux, 92%; (ii) (a) TFA–H_2_O (9 : 1), DCM, 2 h, RT, 85%, (b) (i-PrCO)_2_O, Py, DMAP, 5 h, RT, 87%; (iii) (a) TMSBr, MeCN, 1 h, RT, (b) MeOH, MeONa, then Dowex50W8-Et_3_NH^+^, quant; (iv) Im_2_CO, DMF, then (Bu_3_NH)_3_PO_4_, 16 h, RT, ion-exchange chromatography, 15%.

We attempted to use the *Af*UAP1 structural/mechanistic insights generated here to design a mechanism-inspired substrate analogue inhibitor. The *Af*UAP1–UDP-GlcNAc–PPi Michaelis complex (Fig. 4) and the proposed reaction mechanism (Fig. 5) reveal the direct attack of the phosphate group of GlcNAc-1P on UTP α,β-phosphodiester bond leading to the formation of the products UDP-GlcNAc and PPi. This suggests that an α,β-methylenebisphosphonate analogue of UTP (*me*UTP) in which the scissile α,β-pyrophosphate bond is replaced with a methylenebisphosphonate could inhibit this forward reaction (Fig. 6A). Synthesis of this analogue was initiated with the preparation of a fully protected UDP methylenebisphosphonate analogue **2** (Scheme 1) using a variant of Mitsunobu esterification of 2′,3′-*O*-isopropylideneuridine with methylenebisphosphonic acid trimethylester as a key step (Scheme 1).^26^ In these conditions, the target compound was obtained in 90% yield on a gram scale after conventional chromatography. This novel and highly effective method offers valuable advantages over the reported procedure for direct synthesis of nucleoside 5′-methylenebisphosphonates, which requires large excess of methylenebisphosphonic acid tetrachloride^32^ to be used to minimize formation of symmetrical and/or cyclic by-products. According to the initial synthetic plan, compound **2** was converted into the 2′,3′-di-*O*-isobutyrate derivative **3** in a two-step protective group exchange (Scheme 1). Unfortunately, all attempts to perform selective mono demethylation at the terminal phosphorous atom of the methylene bisphosphonate moiety in **3** to ensure regioselective formation of the triphosphate bond were unsuccessful. After some experimentation, conventional phosphorylation of the fully deprotected α,β-methylenebisphosphonate UDP analogue **4** was proven to be the easiest way to reach the target molecule. To this end, stepwise removal of the protecting groups from **3** gave rise to **4** in near quantitative yield. Finally, phosphorylation of **4** with *N*,*N*′-carbonyldiimidazole and tributylammonium phosphate in DMF for 16 h furnished the desired compound **5** or *me*UTP (Fig. 6A) in a modest 15% yield after ion-exchange chromatography. The structure of compound **5** was ultimately confirmed by ^31^P NMR, with the spectrum showing the characteristic pattern of three different phosphorous signals as doublet at *δ* 17.8 (*J* = 9.3), doublet of doublets at *δ* 6.18 (*J* = 23.6, 9.3), and doublet at *δ* −9.25 (*J* = 23.6) corresponding to γ, β, and α phosphorous atoms only compatible with the linear phosphate-methylenebisphosphonate structural motif. The HPLC profile of the purified compound **5** (Fig. S3A, ESI†) reveals the content of the main substance no less than 98% along with a contamination (2%) corresponding to *me*UDP.

Initial experiments showed that *Af*UAP1 was unable to utilize the *me*UTP as substrate. Therefore, potential inhibition by this compound was studied by an HPAEC assay, allowing us to follow the conversion of UTP to UDP-GlcNAc. Using this approach, it was observed that at increasing concentrations of the *me*UTP, less UTP is consumed while the amount of UDP-GlcNAc produced decreases, suggesting that *me*UTP is an *Af*UAP1 inhibitor (Fig. 6B). We have noted that while the concentration of the inhibitor increases, a peak at *R*_t_ = 3.33 min corresponding to *me*UDP impurity also increases steadily. At 200 µmol concentration of the inhibitor the estimated concentration of *me*UDP reaches about 10–15 µmol, which was deduced from the comparison of the areas under the *me*UDP, UDP-GlcNAc, and UTP peaks. This estimation exceeds the calculated concentration of *me*UDP impurity based on 2% contamination of the *me*UTP 2 to 3 folds. It is not clear if this is a result of enzymatic conversion of *me*UTP or whether the *me*UDP level increases due to partial hydrolysis of the inhibitor in mildly basic conditions of the reaction quench/sample processing. Additionally, a peak at *R*_t_ = 3.80 min was identified as UDP and probably originates from the similar decay of UTP during processing of the enzymatic reaction. To rule out whether *me*UDP is the source of the observed enzyme inhibition, we showed that *Af*UAP1 is not inhibited by 500 µM *me*UDP (Fig. S3B and C, ESI†), eliminating this possibility.

The data show that the α,β-methylenebisphosphonate UTP analogue **5** (*me*UTP) inhibits *Af*UAP1 activity with an IC_50_ of 115 ± 40 µM (Fig. 6C), representing one of the few examples of a rationally designed inhibitor of this class of enzyme. Although this compound is likely to be an aspecific inhibitor of UAP1s and other UTP-dependent enzymes, further structural modifications guided by small differences between the human and the fungal enzymes^13^ may lead to development of selective inhibitors of fungal UAP1. The possibility of inhibition of specific UAP1s, despite high sequence conservation in this family, has recently been demonstrated with a selective UTP-competitive inhibitor of *T. brucei* UAP1 (*Tb*UAP1).^10^ This inhibitor was shown to compete with UTP, binding with a *K*_D_ of 9.3 µM (IC_50_ of 37 ± 4 µM), making it more potent than the *me*UTP.^10^ In addition, the *Tb*UAP1 inhibitor was selective towards this parasite enzyme because it binds to a unique allosteric site on *Tb*UAP1 that is not present in either the human or the fungal UAP1.

## Concluding remarks

The structural data described here provide detailed snapshots of the enzyme in complex with substrates/products, revealing for the first time the key role that the magnesium ion plays in stabilizing the Michaelis complex of pyrophosphorolysis, aligning the reactants for a direct attack by the pyrophosphate group, in parallel with significant conformational changes in the enzyme. We also show that eukaryotic UAP1s depend solely on one magnesium ion unlike the bacterial GlmUs that require two magnesium ions for catalysis. Conserved amino acids interacting with the negative charges on the phosphates were shown to be important for full catalytic activity and in particular we demonstrated that eukaryotic UAP1s use a conserved lysine (Lys437 in *Af*UAP1) as a key electron withdrawer to increase the electrophilic character of the α-phosphorous of either UTP or UDP-GlcNAc in order to promote the forward or reverse catalytic reaction respectively. Furthermore, we have reported for the first time that an α,β-methylenebisphosphonate UTP analogue (*me*UTP) inhibits *Af*UAP1 with an IC_50_ of 115 µM. This and its derivatives may also be a useful reagent for the study of the broader family of sugar nucleotide pyrophosphorylases. For example, it could be used in kinetic protein crystallography experiments that could help to trap kinetic states of pyrophosphorylases, and to determine the outcome of inhibiting these enzymes in appropriate cell models. Together, the novel mechanistic insights and the substrate-based inhibitor reported here will aid the future exploitation of this genetically validated enzyme as a potential target for the discovery of novel antifungal compounds.

## Author contribution

D. M. F. v. A., conceived the idea. R. H.-G. and O. G. R. designed the crystallization constructs and solved the crystal structures. R. H.-G. and O. G. R. purified the enzymes, crystallized the complexes, and refined the crystal structures. O. G. R. performed the kinetic experiments. V. B., synthesized the *me*UTP, A. F. cloned the K437R mutant, M. D. U. performed the HPAEC assay, M. A. J. F., R. H.-G., O. G. R. and D. M. F. v. A. wrote the manuscript. All authors read and approved the final manuscript.

## Accession codes

The coordinates of all the structures have been deposited to the PDB with the accession codes 6G9V, 6TN3 and 6G9W.

## Conflicts of interest

There are no conflict of interest to declare.

## Supplementary Material

CB-001-C9CB00017H-s001

CB-001-C9CB00017H-s002

CB-001-C9CB00017H-s003

CB-001-C9CB00017H-s004

CB-001-C9CB00017H-s005

CB-001-C9CB00017H-s006

CB-001-C9CB00017H-s007
