# Supplementary material for: A mechanism-inspired UDP-N-acetylglucosamine pyrophosphorylase inhibitor
Source: RSC Chem Biol. 2020 Mar 24;1(1):13–25. doi: 10.1039/c9cb00017h (PMC8386105; doi:10.1039/c9cb00017h)
Supplement: CB-001-C9CB00017H-s004 [file CB-001-C9CB00017H-s004.pdf]

Full wwPDB X-ray Structure Validation Report (i)

Apr 18, 2018 – 02:39 PM BST

PDB ID	:	6G9V
Title	:	Crystal structure of Aspergillus fumigatus UDP-N-acetylglucosamine pyro phosphorylase(AfUAP1) in complex with UDPGlcNAc, pyrophosphate and Mg2+
Deposited on	:	2018-04-11
Resolution	:	1.75 Å(reported)

CONFIDENTIAL VALIDATION REPORT

This is a Full wwPDB X-ray Structure Validation Report.

This report is produced by the wwPDB biocuration pipeline after annotation of the structure.

We welcome your comments at *validation@mail.wwpdb.org*
A user guide is available at
https://www.wwpdb.org/validation/2017/XrayValidationReportHelp
with specific help available everywhere you see the (i) symbol.

The following versions of software and data (see references (i)) were used in the production of this report:

MolProbity	:	4.02b-467
Mogul	:	1.7.3 (157068), CSD as539be (2018)
Xtriage (Phenix)	:	1.13
EDS	:	rb-20031021
Percentile statistics	:	20171227.v01 (using entries in the PDB archive December 27th 2017)
Refmac	:	5.8.0158
CCP4	:	7.0 (Gargrove)
Ideal geometry (proteins)	:	Engh & Huber (2001)
Ideal geometry (DNA, RNA)	:	Parkinson et al. (1996)
Validation Pipeline (wwPDB-VP)	:	rb-20031021

1 Overall quality at a glance (i)

The following experimental techniques were used to determine the structure:
X-RAY DIFFRACTION

The reported resolution of this entry is 1.75 Å.

Percentile scores (ranging between 0-100) for global validation metrics of the entry are shown in the following graphic. The table shows the number of entries on which the scores are based.

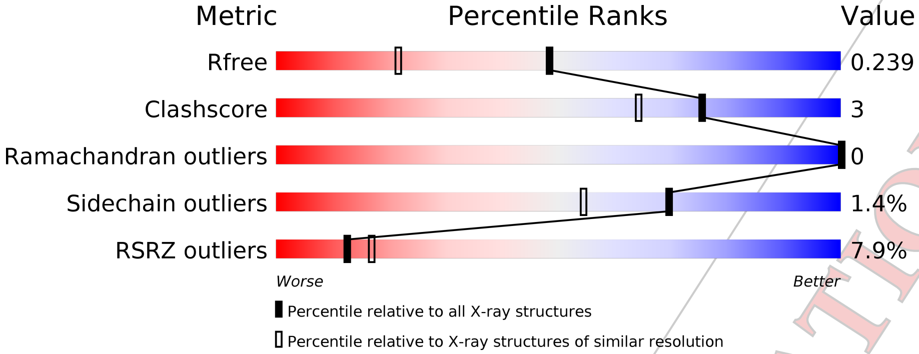

Metric	Whole archive (#Entries)	Similar resolution (#Entries, resolution range(Å))
R_{free}	111664	1952 (1.76-1.76)
Clashscore	122126	2072 (1.76-1.76)
Ramachandran outliers	120053	2050 (1.76-1.76)
Sidechain outliers	120020	2050 (1.76-1.76)
RSRZ outliers	108989	1913 (1.76-1.76)

The table below summarises the geometric issues observed across the polymeric chains and their fit to the electron density. The red, orange, yellow and green segments on the lower bar indicate the fraction of residues that contain outliers for >=3, 2, 1 and 0 types of geometric quality criteria. A grey segment represents the fraction of residues that are not modelled. The numeric value for each fraction is indicated below the corresponding segment, with a dot representing fractions <=5% The upper red bar (where present) indicates the fraction of residues that have poor fit to the electron density. The numeric value is given above the bar.

Mol	Chain	Length	Quality of chain
1	A	509	10% / 85% 5% • 9%
1	B	509	4% / 83% 8% 9%

CONFIDENTIAL VALIDATION REPORT

2 Entry composition (i)

There are 5 unique types of molecules in this entry. The entry contains 7902 atoms, of which 0 are hydrogens and 0 are deuteriums.

In the tables below, the ZeroOcc column contains the number of atoms modelled with zero occupancy, the AltConf column contains the number of residues with at least one atom in alternate conformation and the Trace column contains the number of residues modelled with at most 2 atoms.

- Molecule 1 is a protein called UDP-N-acetylglucosamine pyrophosphorylase.

Mol	Chain	Residues	Atoms					ZeroOcc	AltConf	Trace
			Total	C	N	O	S			
1	A	461	3635	2322	623	676	14	0	4	0
1	B	464	3654	2333	624	685	12	0	1	0

- Molecule 2 is URIDINE-DIPHOSPHATE-N-ACETYLGLUCOSAMINE (three-letter code: UD1) (formula: $C_{17}H_{27}N_3O_{17}P_2$).

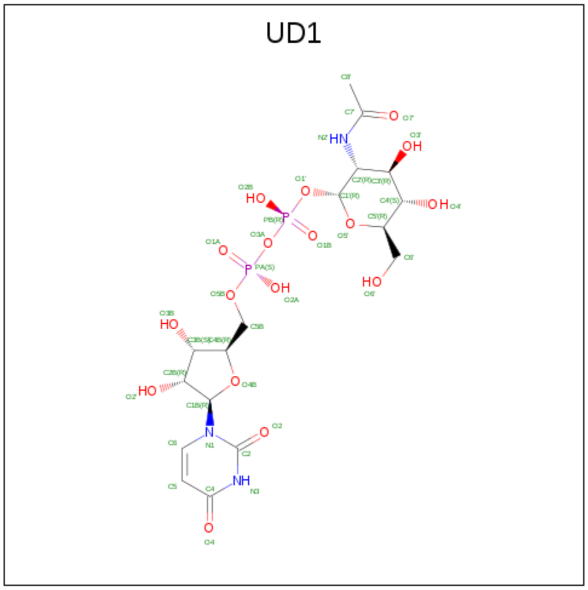

Mol	Chain	Residues	Atoms					ZeroOcc	AltConf
			Total	C	N	O	P		
2	B	1	44	17	3	21	3	0	1

- Molecule 3 is PYROPHOSPHATE (three-letter code: PPV) (formula: $H_4O_7P_2$).

CONFIDENTIAL VALIDATION REPORT

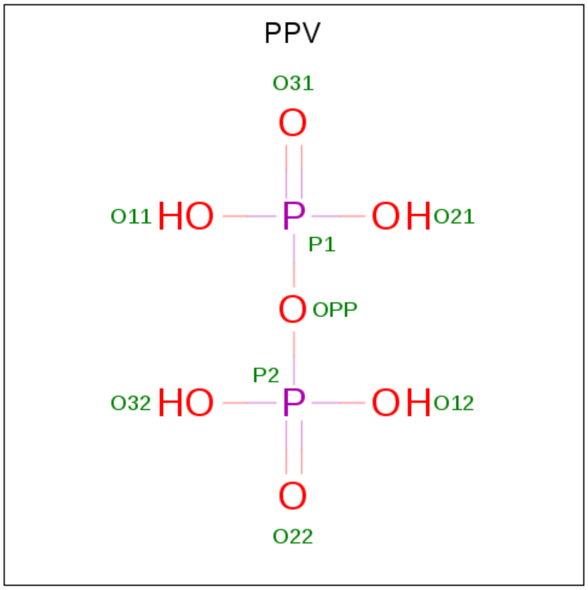

Mol	Chain	Residues	Atoms	ZeroOcc	AltConf
3	B	1	Total O P<br>9 7 2	0	1

- Molecule 4 is MAGNESIUM ION (three-letter code: MG) (formula: Mg).

Mol	Chain	Residues	Atoms	ZeroOcc	AltConf
4	B	1	Total Mg<br>1 1	0	0

- Molecule 5 is water.

Mol	Chain	Residues	Atoms	ZeroOcc	AltConf
5	A	226	Total O<br>226 226	0	0
5	B	333	Total O<br>333 333	0	0

CONFIDENTIAL VALIDATION REPORT

3 Residue-property plots (i)

These plots are drawn for all protein, RNA and DNA chains in the entry. The first graphic for a chain summarises the proportions of the various outlier classes displayed in the second graphic. The second graphic shows the sequence view annotated by issues in geometryand electron density. Residues are color-coded according to the number of geometric quality criteria for which they contain at least one outlier: green = 0, yellow = 1, orange = 2 and red = 3 or more. A red dot above a residue indicates a poor fit to the electron density (RSRZ > 2). Stretches of 2 or more consecutive residues without any outlier are shown as a green connector. Residues present in the sample, but not in the model, are shown in grey.

• Molecule 1: UDP-N-acetylglucosamine pyrophosphorylase

Chain A:

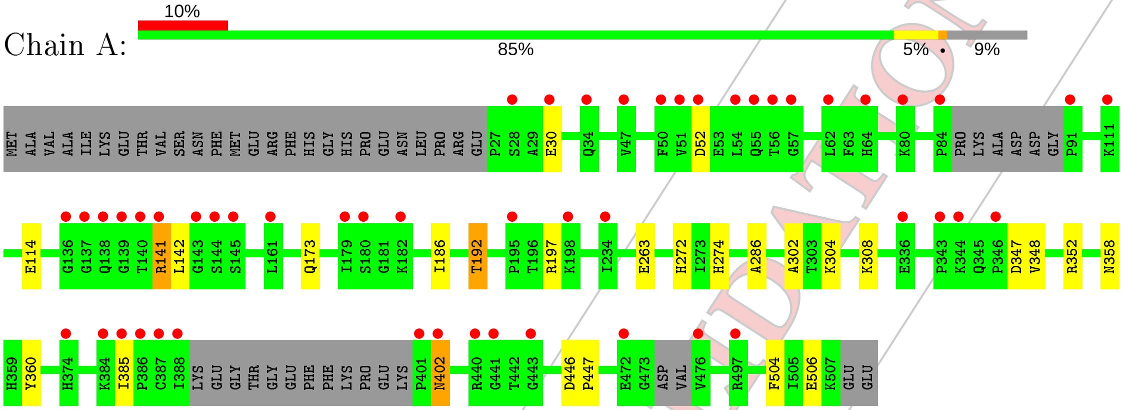

• Molecule 1: UDP-N-acetylglucosamine pyrophosphorylase

Chain B:

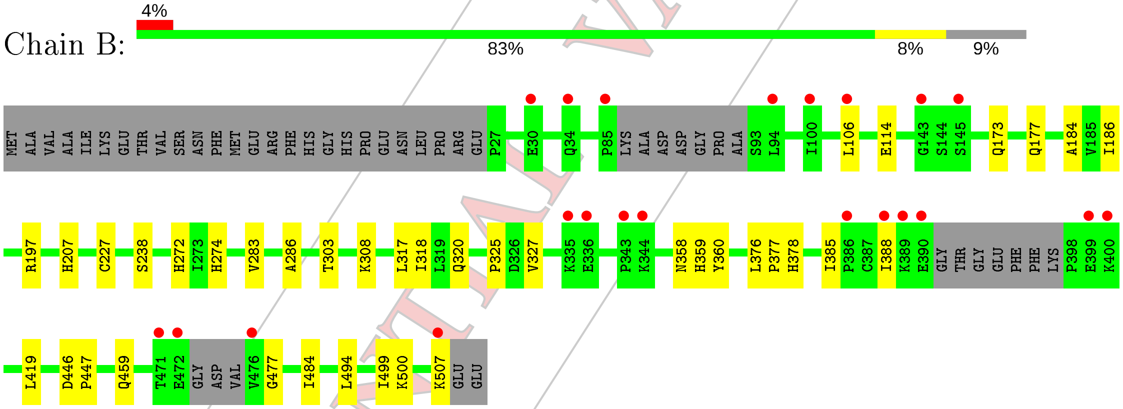

CONFIDENTIAL VALIDATION REPORT

4 Data and refinement statistics (i)

Property	Value	Source
Space group	P 21 21 21	Depositor
Cell constants<br>a, b, c, α, β, γ	55.20Å 139.84Å 144.62Å<br>90.00° 90.00° 90.00°	Depositor
Resolution (Å)	19.97 – 1.75<br>19.97 – 1.75	Depositor<br>EDS
% Data completeness<br>(in resolution range)	100.0 (19.97-1.75)<br>98.7 (19.97-1.75)	Depositor<br>EDS
R_{merge}	0.06	Depositor
R_{sym}	(Not available)	Depositor
$< I/\sigma(I) >$ [1]	1.96 (at 1.74Å)	Xtriage
Refinement program	REFMAC 5.5.0088	Depositor
R, R_{free}	0.207 , 0.240<br>0.207 , 0.239	Depositor<br>DCC
R_{free} test set	1137 reflections (1.01%)	wwPDB-VP
Wilson B-factor (Å^2)	20.1	Xtriage
Anisotropy	0.046	Xtriage
Bulk solvent k_{sol}(e/Å^3), B_{sol}(Å^2)	0.40 , 48.4	EDS
L-test for twinning[2]	$< \|L\| >$ = 0.49, $< L^2 >$ = 0.32	Xtriage
Estimated twinning fraction	0.011 for -h,l,k	Xtriage
F_o,F_c correlation	0.95	EDS
Total number of atoms	7902	wwPDB-VP
Average B, all atoms (Å^2)	24.0	wwPDB-VP

Xtriage's analysis on translational NCS is as follows: *The largest off-origin peak in the Patterson function is 3.58% of the height of the origin peak. No significant pseudotranslation is detected.*

[1] Intensities estimated from amplitudes.

[2] Theoretical values of $< |L| >$, $< L^2 >$ for acentric reflections are 0.5, 0.333 respectively for untwinned datasets, and 0.375, 0.2 for perfectly twinned datasets.

CONFIDENTIAL VALIDATION REPORT

5 Model quality (i)

5.1 Standard geometry (i)

Bond lengths and bond angles in the following residue types are not validated in this section: MG, UD1, PPV

The Z score for a bond length (or angle) is the number of standard deviations the observed value is removed from the expected value. A bond length (or angle) with $|Z| > 5$ is considered an outlier worth inspection. RMSZ is the root-mean-square of all Z scores of the bond lengths (or angles).

Mol	Chain	Bond lengths		Bond angles	
		RMSZ	#$|Z|$ >5	RMSZ	#$|Z|$ >5
1	A	0.48	0/3724	0.54	0/5020
1	B	0.44	0/3731	0.52	0/5030
All	All	0.46	0/7455	0.53	0/10050

There are no bond length outliers.

There are no bond angle outliers.

There are no chirality outliers.

There are no planarity outliers.

5.2 Too-close contacts (i)

In the following table, the Non-H and H(model) columns list the number of non-hydrogen atoms and hydrogen atoms in the chain respectively. The H(added) column lists the number of hydrogen atoms added and optimized by MolProbity. The Clashes column lists the number of clashes within the asymmetric unit, whereas Symm-Clashes lists symmetry related clashes.

Mol	Chain	Non-H	H(model)	H(added)	Clashes	Symm-Clashes
1	A	3635	0	3658	24	0
1	B	3654	0	3668	26	0
2	B	44	0	4	0	0
3	B	9	0	0	0	0
4	B	1	0	0	0	0
5	A	226	0	0	0	0
5	B	333	0	0	3	0
All	All	7902	0	7330	50	0

The all-atom clashscore is defined as the number of clashes found per 1000 atoms (including hydrogen atoms). The all-atom clashscore for this structure is 3.

CONFIDENTIAL VALIDATION REPORT

All (50) close contacts within the same asymmetric unit are listed below, sorted by their clash magnitude.

Atom-1	Atom-2	Interatomic distance (Å)	Clash overlap (Å)
1:A:385:ILE:H	1:A:402:ASN:HD21	1.07	0.98
1:A:274:HIS:HD2	1:A:286:ALA:H	1.23	0.86
1:A:173:GLN:HE21	1:A:186:ILE:H	1.23	0.84
1:B:173:GLN:HE21	1:B:186:ILE:H	1.21	0.84
1:B:274:HIS:HD2	1:B:286:ALA:H	1.31	0.79
1:A:274:HIS:CD2	1:A:286:ALA:H	2.04	0.74
1:A:402:ASN:HD22	1:A:402:ASN:H	1.34	0.73
1:A:385:ILE:H	1:A:402:ASN:ND2	1.84	0.70
1:B:272:HIS:HD2	1:B:360:TYR:OH	1.76	0.69
1:A:141:ARG:NH2	1:A:141:ARG:HG2	2.10	0.66
1:B:274:HIS:CD2	1:B:286:ALA:H	2.13	0.66
1:B:177:GLN:HE21	1:B:184:ALA:H	1.42	0.66
1:A:272:HIS:HD2	1:A:360:TYR:OH	1.84	0.60
1:B:238:SER:HA	1:B:388:ILE:HD13	1.82	0.60
1:A:446:ASP:HB2	1:A:447:PRO:HD2	1.83	0.60
1:A:141:ARG:HH21	1:A:141:ARG:HG2	1.66	0.60
1:B:274:HIS:CE1	1:B:358:ASN:HD22	2.20	0.60
1:B:303:THR:OG1	1:B:359:HIS:HD2	1.84	0.60
1:B:274:HIS:HE1	1:B:358:ASN:HD22	1.50	0.59
1:B:378:HIS:HE1	5:B:972:HOH:O	1.85	0.58
1:A:173:GLN:NE2	1:A:186:ILE:H	1.98	0.57
1:B:106:LEU:HD12	1:B:106:LEU:H	1.71	0.56
1:B:173:GLN:NE2	1:B:186:ILE:H	1.98	0.56
1:A:274:HIS:HE1	1:A:358:ASN:OD1	1.90	0.55
1:A:446:ASP:HB2	1:A:447:PRO:CD	2.37	0.55
1:A:141:ARG:HH21	1:A:141:ARG:CG	2.23	0.52
1:A:192:THR:HG21	1:A:197:ARG:HA	1.91	0.52
1:A:402:ASN:HD22	1:A:402:ASN:N	2.05	0.52
1:B:459:GLN:HE21	1:B:477:GLY:HA2	1.75	0.52
1:B:325:PRO:HD3	1:B:419:LEU:HG	1.91	0.51
1:A:348:VAL:CG1	1:A:352:ARG:HD3	2.40	0.50
1:B:446:ASP:HB2	1:B:447:PRO:CD	2.43	0.49
1:B:320:GLN:HE21	1:B:419:LEU:HD11	1.79	0.48
1:B:484:ILE:HD11	1:B:507:LYS:HA	1.96	0.47
1:B:308:LYS:HD2	1:B:317:LEU:HD21	1.97	0.47
1:B:227:CYS:HB3	1:B:385:ILE:HD13	1.97	0.47
1:B:283:VAL:HG23	1:B:358:ASN:HD21	1.79	0.46
1:B:494:LEU:HD22	1:B:499:ILE:HD11	1.98	0.46
1:B:318:ILE:HG12	1:B:327:VAL:HG22	1.97	0.46
1:B:207:HIS:HD2	5:B:978:HOH:O	2.00	0.45

Continued on next page...

CONFIDENTIAL VALIDATION REPORT

Continued from previous page...

Atom-1	Atom-2	Interatomic distance (Å)	Clash overlap (Å)
1:A:504:PHE:CZ	1:A:506:GLU:HB2	2.53	0.43
1:A:308:LYS:HB3	1:A:352:ARG:HA	2.01	0.42
1:A:304:LYS:HB2	1:A:358:ASN:HB3	2.02	0.42
1:A:142:LEU:HD12	1:A:142:LEU:O	2.18	0.42
1:B:376:LEU:HA	1:B:377:PRO:HD3	1.94	0.42
1:A:402:ASN:H	1:A:402:ASN:ND2	2.11	0.42
1:A:274:HIS:CD2	1:A:286:ALA:N	2.83	0.41
1:A:302:ALA:HB3	1:A:360:TYR:HB3	2.02	0.41
1:B:320:GLN:NE2	1:B:419:LEU:HD11	2.35	0.41
1:B:272:HIS:HE1	5:B:701:HOH:O	2.04	0.40

There are no symmetry-related clashes.

5.3 Torsion angles (i)

5.3.1 Protein backbone (i)

In the following table, the Percentiles column shows the percent Ramachandran outliers of the chain as a percentile score with respect to all X-ray entries followed by that with respect to entries of similar resolution.

The Analysed column shows the number of residues for which the backbone conformation was analysed, and the total number of residues.

Mol	Chain	Analysed	Favoured	Allowed	Outliers	Percentiles	
1	A	457/509 (90%)	449 (98%)	8 (2%)	0	100	100
1	B	457/509 (90%)	452 (99%)	5 (1%)	0	100	100
All	All	914/1018 (90%)	901 (99%)	13 (1%)	0	100	100

There are no Ramachandran outliers to report.

5.3.2 Protein sidechains (i)

In the following table, the Percentiles column shows the percent sidechain outliers of the chain as a percentile score with respect to all X-ray entries followed by that with respect to entries of similar resolution.

The Analysed column shows the number of residues for which the sidechain conformation was analysed, and the total number of residues.

CONFIDENTIAL VALIDATION REPORT

Mol	Chain	Analysed	Rotameric	Outliers	Percentiles	
1	A	394/431 (91%)	386 (98%)	8 (2%)	58	36
1	B	396/431 (92%)	393 (99%)	3 (1%)	83	74
All	All	790/862 (92%)	779 (99%)	11 (1%)	69	54

All (11) residues with a non-rotameric sidechain are listed below:

Mol	Chain	Res	Type
1	A	30	GLU
1	A	52	ASP
1	A	114	GLU
1	A	141	ARG
1	A	192	THR
1	A	263	GLU
1	A	347	ASP
1	A	402	ASN
1	B	114	GLU
1	B	197	ARG
1	B	500	LYS

Some sidechains can be flipped to improve hydrogen bonding and reduce clashes. All (21) such sidechains are listed below:

Mol	Chain	Res	Type
1	A	115	GLN
1	A	163	GLN
1	A	173	GLN
1	A	177	GLN
1	A	272	HIS
1	A	274	HIS
1	A	359	HIS
1	A	378	HIS
1	A	402	ASN
1	B	34	GLN
1	B	163	GLN
1	B	173	GLN
1	B	177	GLN
1	B	207	HIS
1	B	272	HIS
1	B	274	HIS
1	B	320	GLN
1	B	358	ASN
1	B	359	HIS

Continued on next page...

CONFIDENTIAL VALIDATION REPORT

Continued from previous page...

Mol	Chain	Res	Type
1	B	378	HIS
1	B	459	GLN

5.3.3 RNA (i)

There are no RNA molecules in this entry.

5.4 Non-standard residues in protein, DNA, RNA chains (i)

There are no non-standard protein/DNA/RNA residues in this entry.

5.5 Carbohydrates (i)

There are no carbohydrates in this entry.

5.6 Ligand geometry (i)

Of 4 ligands modelled in this entry, 1 is monoatomic - leaving 3 for Mogul analysis.

In the following table, the Counts columns list the number of bonds (or angles) for which Mogul statistics could be retrieved, the number of bonds (or angles) that are observed in the model and the number of bonds (or angles) that are defined in the Chemical Component Dictionary. The Link column lists molecule types, if any, to which the group is linked. The Z score for a bond length (or angle) is the number of standard deviations the observed value is removed from the expected value. A bond length (or angle) with $|Z| > 2$ is considered an outlier worth inspection. RMSZ is the root-mean-square of all Z scores of the bond lengths (or angles).

Mol	Type	Chain	Res	Link	Bond lengths			Bond angles		
					Counts	RMSZ	$\#\|Z\| > 2$	Counts	RMSZ	$\#\|Z\| > 2$
2	UD1	B	601[A]	4	33,41,41	1.52	3 (9%)	45,62,62	1.48	2 (4%)
2	UD1	B	601[B]	4	33,41,41	1.51	3 (9%)	45,62,62	1.49	3 (6%)
3	PPV	B	602[B]	4	8,8,8	1.23	0	9,13,13	1.07	1 (11%)

In the following table, the Chirals column lists the number of chiral outliers, the number of chiral centers analysed, the number of these observed in the model and the number defined in the Chemical Component Dictionary. Similar counts are reported in the Torsion and Rings columns. '-' means no outliers of that kind were identified.

Mol	Type	Chain	Res	Link	Chirals	Torsions	Rings
2	UD1	B	601[A]	4	-	0/22/63/63	0/3/3/3

Continued on next page...

CONFIDENTIAL VALIDATION REPORT

Continued from previous page...

Mol	Type	Chain	Res	Link	Chirals	Torsions	Rings
2	UD1	B	601[B]	4	-	0/22/63/63	0/3/3/3
3	PPV	B	602[B]	4	-	0/6/6/6	0/0/0/0

All (6) bond length outliers are listed below:

Mol	Chain	Res	Type	Atoms	Z	Observed(Å)	Ideal(Å)
2	B	601[A]	UD1	O4B-C1B	3.31	1.45	1.41
2	B	601[B]	UD1	O4B-C1B	3.31	1.45	1.41
2	B	601[A]	UD1	C4-N3	4.49	1.41	1.33
2	B	601[B]	UD1	C4-N3	4.49	1.41	1.33
2	B	601[A]	UD1	C6-N1	4.83	1.42	1.35
2	B	601[B]	UD1	C6-N1	4.83	1.42	1.35

All (6) bond angle outliers are listed below:

Mol	Chain	Res	Type	Atoms	Z	Observed(^{o})	Ideal(^{o})
3	B	602[B]	PPV	P2-OPP-P1	-2.57	123.99	132.63
2	B	601[A]	UD1	C4'-C3'-C2'	-2.14	107.16	110.33
2	B	601[B]	UD1	C4'-C3'-C2'	-2.14	107.16	110.33
2	B	601[B]	UD1	O3A-PB-O1'	-2.00	98.43	102.53
2	B	601[A]	UD1	C4-N3-C2	8.21	121.20	114.14
2	B	601[B]	UD1	C4-N3-C2	8.21	121.20	114.14

There are no chirality outliers.

There are no torsion outliers.

There are no ring outliers.

No monomer is involved in short contacts.

5.7 Other polymers (i)

There are no such residues in this entry.

5.8 Polymer linkage issues (i)

There are no chain breaks in this entry.

CONFIDENTIAL VALIDATION REPORT

6 Fit of model and data (i)

6.1 Protein, DNA and RNA chains (i)

In the following table, the column labelled '#RSRZ> 2' contains the number (and percentage) of RSRZ outliers, followed by percent RSRZ outliers for the chain as percentile scores relative to all X-ray entries and entries of similar resolution. The OWAB column contains the minimum, median, 95th percentile and maximum values of the occupancy-weighted average B-factor per residue. The column labelled 'Q< 0.9' lists the number of (and percentage) of residues with an average occupancy less than 0.9.

Mol	Chain	Analysed	<RSRZ>	#RSRZ>2			OWAB(Å^2)	Q<0.9
1	A	461/509 (90%)	0.64	51 (11%)	5	7	13, 25, 41, 48	0
1	B	464/509 (91%)	0.24	22 (4%)	31	37	10, 20, 37, 47	0
All	All	925/1018 (90%)	0.44	73 (7%)	12	17	10, 22, 40, 48	0

All (73) RSRZ outliers are listed below:

Mol	Chain	Res	Type	RSRZ
1	A	139	GLY	6.7
1	A	386	PRO	6.3
1	A	401	PRO	5.9
1	A	140	THR	5.7
1	A	143	GLY	4.9
1	B	343	PRO	4.8
1	A	441	GLY	4.7
1	A	179	ILE	4.6
1	A	388	ILE	4.4
1	B	388	ILE	4.2
1	A	343	PRO	4.2
1	A	374	HIS	4.2
1	B	85	PRO	4.0
1	A	55	GLN	4.0
1	A	91	PRO	4.0
1	A	384	LYS	3.9
1	A	52	ASP	3.9
1	A	387	CYS	3.9
1	A	56	THR	3.6
1	A	385	ILE	3.6
1	B	344	LYS	3.5
1	B	472	GLU	3.4
1	B	389	LYS	3.3
1	A	141	ARG	3.3

Continued on next page...

CONFIDENTIAL VALIDATION REPORT

Continued from previous page...

Mol	Chain	Res	Type	RSRZ
1	B	507	LYS	3.3
1	B	145	SER	3.2
1	B	30	GLU	3.2
1	A	138	GLN	3.2
1	A	476	VAL	3.1
1	A	180	SER	3.1
1	A	51	VAL	3.1
1	A	136	GLY	3.1
1	A	198	LYS	3.0
1	A	440	ARG	2.9
1	B	386	PRO	2.7
1	A	443	GLY	2.7
1	A	472	GLU	2.7
1	A	161	LEU	2.7
1	A	145	SER	2.7
1	B	143	GLY	2.7
1	B	94	LEU	2.7
1	A	84	PRO	2.7
1	A	195	PRO	2.7
1	B	471	THR	2.6
1	A	64[A]	HIS	2.6
1	A	182	LYS	2.6
1	A	111	LYS	2.6
1	A	144	SER	2.5
1	A	402	ASN	2.4
1	B	106	LEU	2.4
1	A	57	GLY	2.4
1	A	137	GLY	2.4
1	A	34	GLN	2.4
1	A	28	SER	2.4
1	A	80	LYS	2.4
1	A	47	VAL	2.3
1	B	100	ILE	2.3
1	A	50	PHE	2.3
1	B	335	LYS	2.2
1	B	399	GLU	2.2
1	A	336	GLU	2.2
1	B	400	LYS	2.2
1	A	344	LYS	2.2
1	A	30	GLU	2.1
1	B	336	GLU	2.1
1	A	346	PRO	2.1

Continued on next page...

CONFIDENTIAL VALIDATION REPORT

Continued from previous page...

Mol	Chain	Res	Type	RSRZ
1	A	62	LEU	2.1
1	A	497	ARG	2.1
1	B	34	GLN	2.1
1	A	54	LEU	2.1
1	B	390	GLU	2.1
1	A	234	ILE	2.1
1	B	476	VAL	2.0

6.2 Non-standard residues in protein, DNA, RNA chains (i)

There are no non-standard protein/DNA/RNA residues in this entry.

6.3 Carbohydrates (i)

There are no carbohydrates in this entry.

6.4 Ligands (i)

In the following table, the Atoms column lists the number of modelled atoms in the group and the number defined in the chemical component dictionary. The B-factors column lists the minimum, median, 95th percentile and maximum values of B factors of atoms in the group. The column labelled 'Q< 0.9' lists the number of atoms with occupancy less than 0.9.

Mol	Type	Chain	Res	Atoms	RSCC	RSR	B-factors($Å^2$)	Q<0.9
3	PPV	B	602[B]	9/9	0.93	0.19	14,17,19,19	9
2	UD1	B	601[A]	39/39	0.96	0.08	12,16,19,20	5
2	UD1	B	601[B]	39/39	0.96	0.08	12,16,20,21	5
4	MG	B	603	1/1	0.96	0.12	33,33,33,33	0

6.5 Other polymers (i)

There are no such residues in this entry.

CONFIDENTIAL VALIDATION REPORT
