# Supplementary material for: A mechanism-inspired UDP-N-acetylglucosamine pyrophosphorylase inhibitor
Source: RSC Chem Biol. 2020 Mar 24;1(1):13–25. doi: 10.1039/c9cb00017h (PMC8386105; doi:10.1039/c9cb00017h)
Supplement: CB-001-C9CB00017H-s005 [file CB-001-C9CB00017H-s005.pdf]

Full wwPDB X-ray Structure Validation Report (i)

Apr 12, 2018 – 11:33 AM BST

PDB ID	:	6G9W
Title	:	Crystal Structure of Aspergillus fumigatus UDP-N-acetylglucosamine pyrophosphorylase in complex with UTP
Deposited on	:	2018-04-11
Resolution	:	2.40 Å(reported)

This is a Full wwPDB X-ray Structure Validation Report.

This report is produced by the wwPDB biocuration pipeline after annotation of the structure.

We welcome your comments at *validation@mail.wwpdb.org*
A user guide is available at
https://www.wwpdb.org/validation/2017/XrayValidationReportHelp
with specific help available everywhere you see the (i) symbol.

CONFIDENTIAL VALIDATION REPORT

The following versions of software and data (see references (i)) were used in the production of this report:

MolProbity	:	4.02b-467
Mogul	:	1.7.3 (157068), CSD as539be (2018)
Xtriage (Phenix)	:	1.13
EDS	:	rb-20031021
Percentile statistics	:	20171227.v01 (using entries in the PDB archive December 27th 2017)
Refmac	:	5.8.0158
CCP4	:	7.0 (Gargrove)
Ideal geometry (proteins)	:	Engh & Huber (2001)
Ideal geometry (DNA, RNA)	:	Parkinson et al. (1996)
Validation Pipeline (wwPDB-VP)	:	rb-20031021

1 Overall quality at a glance (i)

The following experimental techniques were used to determine the structure:
X-RAY DIFFRACTION

The reported resolution of this entry is 2.40 Å.

Percentile scores (ranging between 0-100) for global validation metrics of the entry are shown in the following graphic. The table shows the number of entries on which the scores are based.

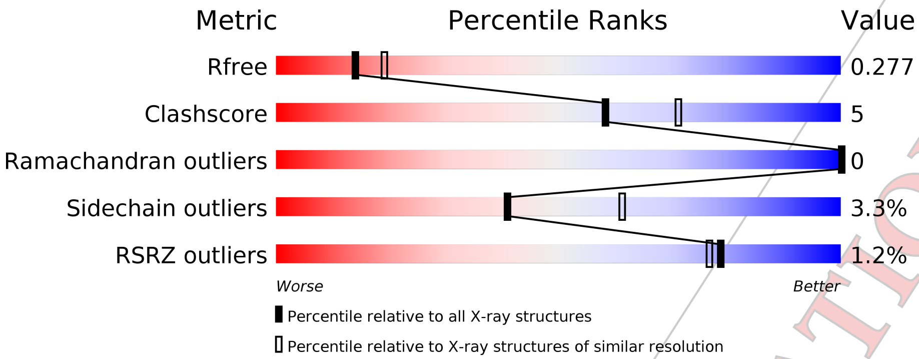

Metric	Whole archive (#Entries)	Similar resolution (#Entries, resolution range(Å))
R_{free}	111664	3481 (2.40-2.40)
Clashscore	122126	3956 (2.40-2.40)
Ramachandran outliers	120053	3897 (2.40-2.40)
Sidechain outliers	120020	3898 (2.40-2.40)
RSRZ outliers	108989	3386 (2.40-2.40)

The table below summarises the geometric issues observed across the polymeric chains and their fit to the electron density. The red, orange, yellow and green segments on the lower bar indicate the fraction of residues that contain outliers for >=3, 2, 1 and 0 types of geometric quality criteria. A grey segment represents the fraction of residues that are not modelled. The numeric value for each fraction is indicated below the corresponding segment, with a dot representing fractions <=5% The upper red bar (where present) indicates the fraction of residues that have poor fit to the electron density. The numeric value is given above the bar.

Mol	Chain	Length	Quality of chain
1	A	509	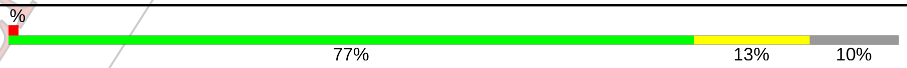
1	B	509	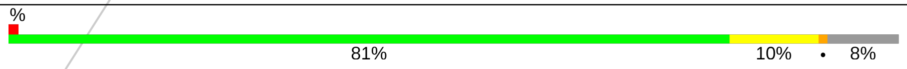

CONFIDENTIAL VALIDATION REPORT

2 Entry composition (i)

There are 3 unique types of molecules in this entry. The entry contains 7507 atoms, of which 0 are hydrogens and 0 are deuteriums.

In the tables below, the ZeroOcc column contains the number of atoms modelled with zero occupancy, the AltConf column contains the number of residues with at least one atom in alternate conformation and the Trace column contains the number of residues modelled with at most 2 atoms.

- Molecule 1 is a protein called UDP-N-acetylglucosamine pyrophosphorylase.

Mol	Chain	Residues	Atoms					ZeroOcc	AltConf	Trace
			Total	C	N	O	S			
1	A	460	3616	2308	618	676	14	0	1	0
1	B	468	3684	2356	628	687	13	0	1	0

- Molecule 2 is URIDINE 5'-TRIPHOSPHATE (three-letter code: UTP) (formula: $C_9H_{15}N_2O_{15}P_3$).

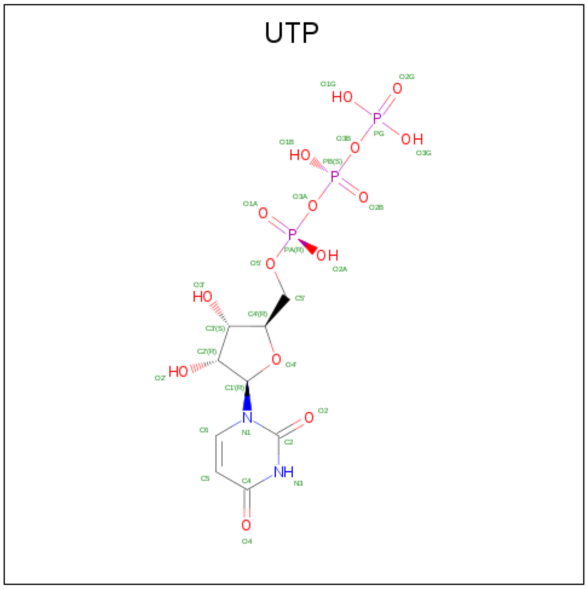

Mol	Chain	Residues	Atoms					ZeroOcc	AltConf
			Total	C	N	O	P		
2	A	1	58	18	4	30	6	0	1
2	B	1	29	9	2	15	3	0	0

- Molecule 3 is water.

CONFIDENTIAL VALIDATION REPORT

Mol	Chain	Residues	Atoms	ZeroOcc	AltConf
3	A	50	Total O<br>50 50	0	0
3	B	70	Total O<br>70 70	0	0

CONFIDENTIAL VALIDATION REPORT

3 Residue-property plots ⓘ

These plots are drawn for all protein, RNA and DNA chains in the entry. The first graphic for a chain summarises the proportions of the various outlier classes displayed in the second graphic. The second graphic shows the sequence view annotated by issues in geometryand electron density. Residues are color-coded according to the number of geometric quality criteria for which they contain at least one outlier: green = 0, yellow = 1, orange = 2 and red = 3 or more. A red dot above a residue indicates a poor fit to the electron density (RSRZ > 2). Stretches of 2 or more consecutive residues without any outlier are shown as a green connector. Residues present in the sample, but not in the model, are shown in grey.

- Molecule 1: UDP-N-acetylglucosamine pyrophosphorylase

Chain A:

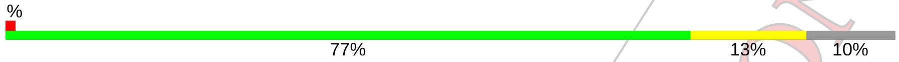

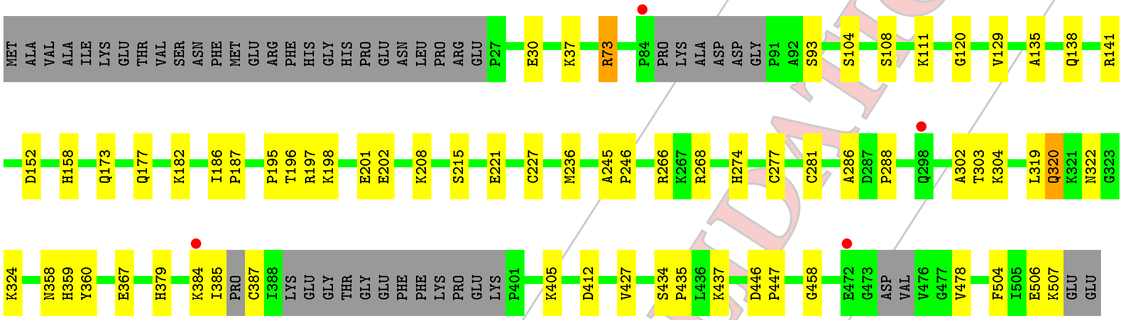

- Molecule 1: UDP-N-acetylglucosamine pyrophosphorylase

Chain B:

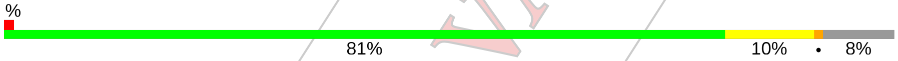

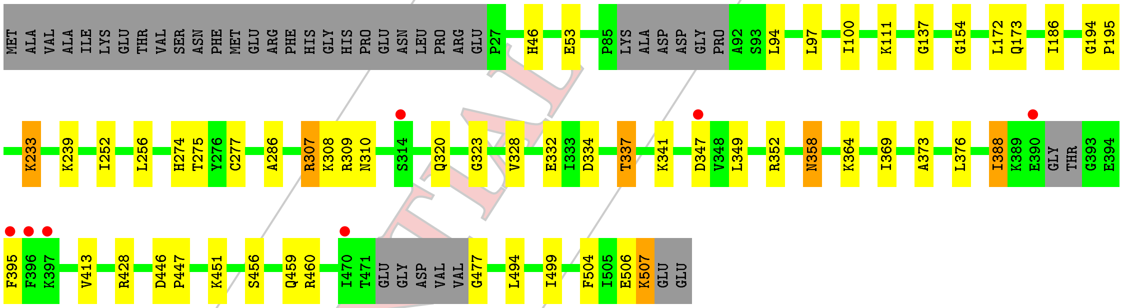

CONFIDENTIAL VALIDATION REPORT

4 Data and refinement statistics (i)

Property	Value	Source
Space group	P 21 21 21	Depositor
Cell constants<br>a, b, c, α, β, γ	56.71Å 139.98Å 146.29Å<br>90.00° 90.00° 90.00°	Depositor
Resolution (Å)	20.00 – 2.40<br>19.92 – 2.40	Depositor<br>EDS
% Data completeness<br>(in resolution range)	99.1 (20.00-2.40)<br>99.1 (19.92-2.40)	Depositor<br>EDS
R_{merge}	(Not available)	Depositor
R_{sym}	(Not available)	Depositor
$< I/\sigma(I) >$ [1]	2.92 (at 2.41Å)	Xtriage
Refinement program	REFMAC 5.2.0019	Depositor
R, R_{free}	0.227 , 0.279<br>0.226 , 0.277	Depositor<br>DCC
R_{free} test set	942 reflections (2.05%)	wwPDB-VP
Wilson B-factor (Å^2)	35.5	Xtriage
Anisotropy	0.042	Xtriage
Bulk solvent k_{sol}(e/Å^3), B_{sol}(Å^2)	0.33 , 26.4	EDS
L-test for twinning[2]	$< \|L\| > = 0.50$, $< L^2 > = 0.33$	Xtriage
Estimated twinning fraction	0.008 for -h,l,k	Xtriage
F_o,F_c correlation	0.92	EDS
Total number of atoms	7507	wwPDB-VP
Average B, all atoms (Å^2)	34.0	wwPDB-VP

Xtriage's analysis on translational NCS is as follows: *The largest off-origin peak in the Patterson function is 3.72% of the height of the origin peak. No significant pseudotranslation is detected.*

CONFIDENTIAL VALIDATION REPORT

[1]Intensities estimated from amplitudes.

[2]Theoretical values of $< |L| >$, $< L^2 >$ for acentric reflections are 0.5, 0.333 respectively for untwinned datasets, and 0.375, 0.2 for perfectly twinned datasets.

5 Model quality (i)

5.1 Standard geometry (i)

Bond lengths and bond angles in the following residue types are not validated in this section: UTP

The Z score for a bond length (or angle) is the number of standard deviations the observed value is removed from the expected value. A bond length (or angle) with $|Z| > 5$ is considered an outlier worth inspection. RMSZ is the root-mean-square of all Z scores of the bond lengths (or angles).

Mol	Chain	Bond lengths		Bond angles	
		RMSZ	#$\|Z\|$ >5	RMSZ	#$\|Z\|$ >5
1	A	0.49	0/3693	0.57	0/4976
1	B	0.54	0/3766	0.62	0/5076
All	All	0.52	0/7459	0.59	0/10052

There are no bond length outliers.

There are no bond angle outliers.

There are no chirality outliers.

There are no planarity outliers.

5.2 Too-close contacts (i)

In the following table, the Non-H and H(model) columns list the number of non-hydrogen atoms and hydrogen atoms in the chain respectively. The H(added) column lists the number of hydrogen atoms added and optimized by MolProbity. The Clashes column lists the number of clashes within the asymmetric unit, whereas Symm-Clashes lists symmetry related clashes.

Mol	Chain	Non-H	H(model)	H(added)	Clashes	Symm-Clashes
1	A	3616	0	3627	40	0
1	B	3684	0	3698	37	0
2	A	58	0	22	4	0
2	B	29	0	11	6	0
3	A	50	0	0	0	0
3	B	70	0	0	1	0
All	All	7507	0	7358	77	0

The all-atom clashscore is defined as the number of clashes found per 1000 atoms (including hydrogen atoms). The all-atom clashscore for this structure is 5.

All (77) close contacts within the same asymmetric unit are listed below, sorted by their clash

CONFIDENTIAL VALIDATION REPORT

magnitude.

Atom-1	Atom-2	Interatomic distance (Å)	Clash overlap (Å)
1:A:73:ARG:HH11	1:A:73:ARG:HG2	1.20	1.06
1:B:277[A]:CYS:SG	2:B:601:UTP:O3'	2.08	0.91
1:B:173:GLN:HE21	1:B:186:ILE:H	1.17	0.89
1:B:334:ASP:HB2	1:B:337:THR:HG23	1.55	0.88
1:B:274:HIS:HE1	1:B:358:ASN:HD21	1.23	0.83
1:A:173:GLN:HE21	1:A:186:ILE:H	1.26	0.83
1:A:73:ARG:HH11	1:A:73:ARG:CG	2.00	0.73
1:B:173:GLN:NE2	1:B:186:ILE:H	1.88	0.71
1:B:274:HIS:HD2	1:B:286:ALA:H	1.40	0.69
1:B:274:HIS:HE1	1:B:358:ASN:ND2	1.92	0.68
1:B:307:ARG:HD2	1:B:352:ARG:HD2	1.76	0.68
1:B:446:ASP:HB2	1:B:447:PRO:CD	2.25	0.66
1:A:274:HIS:HD2	1:A:286:ALA:H	1.43	0.65
1:A:173:GLN:NE2	1:A:186:ILE:H	1.95	0.65
1:A:379:HIS:HE1	1:A:412:ASP:OD2	1.79	0.64
1:A:274:HIS:CD2	1:A:286:ALA:H	2.16	0.63
1:A:177:GLN:HG3	1:A:182:LYS:O	1.99	0.62
1:B:308:LYS:HE2	1:B:310:ASN:O	2.00	0.62
1:B:277[A]:CYS:SG	2:B:601:UTP:H4'	2.40	0.60
1:B:459:GLN:HE21	1:B:477:GLY:HA2	1.67	0.60
1:B:494:LEU:HD22	1:B:499:ILE:HD11	1.85	0.58
1:B:137:GLY:HA3	2:B:601:UTP:H2'	1.86	0.57
1:B:328:VAL:HG13	1:B:332:GLU:HB2	1.87	0.56
1:A:141:ARG:HG2	2:A:601[A]:UTP:O1G	2.05	0.56
1:B:446:ASP:HB2	1:B:447:PRO:HD2	1.85	0.56
1:B:388:ILE:HG13	1:B:395:PHE:HA	1.90	0.54
1:B:376:LEU:HD21	1:B:413:VAL:HG12	1.91	0.53
1:A:73:ARG:NH1	1:A:73:ARG:HG2	2.00	0.53
1:B:428:ARG:NH2	3:B:701:HOH:O	2.42	0.52
1:B:506:GLU:O	1:B:507:LYS:HB3	2.08	0.52
1:A:227:CYS:HB3	1:A:385:ILE:HD13	1.92	0.52
1:A:277:CYS:SG	2:A:601[B]:UTP:H4'	2.50	0.52
1:A:93:SER:H	1:A:322:ASN:ND2	2.08	0.52
1:A:281[C]:CYS:SG	1:A:434:SER:HB3	2.50	0.52
1:A:135:ALA:O	1:A:196:THR:HG21	2.11	0.51
1:B:277[A]:CYS:SG	2:B:601:UTP:C3'	2.98	0.51
1:B:504:PHE:CZ	1:B:506:GLU:HB2	2.46	0.51
1:B:46:HIS:CE1	1:B:233:LYS:HG3	2.45	0.50
1:A:274:HIS:HE1	1:A:358:ASN:OD1	1.94	0.50
1:A:446:ASP:HB2	1:A:447:PRO:CD	2.42	0.50
1:A:197:ARG:NE	1:A:201:GLU:OE2	2.37	0.50

Continued on next page...

CONFIDENTIAL VALIDATION REPORT

Continued from previous page...

Atom-1	Atom-2	Interatomic distance (Å)	Clash overlap (Å)
1:B:252:ILE:O	1:B:256:LEU:HG	2.13	0.49
1:B:53:GLU:HG3	1:B:239:LYS:HE3	1.95	0.49
1:A:245:ALA:CB	1:A:405:LYS:HD3	2.43	0.49
1:A:152:ASP:OD2	1:A:158:HIS:HD2	1.97	0.47
1:B:320:GLN:HE21	1:B:323:GLY:HA2	1.79	0.47
1:B:194:GLY:N	1:B:195:PRO:HD2	2.29	0.46
1:A:320:GLN:HA	1:A:324:LYS:O	2.16	0.46
1:A:173:GLN:HE21	1:A:186:ILE:N	2.02	0.46
1:B:506:GLU:HG3	1:B:507:LYS:N	2.30	0.46
1:A:303:THR:HG22	1:A:359:HIS:CD2	2.51	0.46
1:A:277:CYS:HB3	2:A:601[B]:UTP:O3'	2.16	0.45
1:A:198:LYS:O	1:A:202:GLU:HG3	2.17	0.45
1:A:197:ARG:HD2	1:A:221:GLU:HG3	1.99	0.45
1:A:120:GLY:HA3	1:A:288:PRO:O	2.17	0.44
1:B:274:HIS:CE1	1:B:358:ASN:HD21	2.16	0.44
1:B:277[A]:CYS:HG	2:B:601:UTP:C3'	2.12	0.44
1:A:304:LYS:HE2	1:A:427:VAL:HG11	2.00	0.44
1:A:504:PHE:CZ	1:A:506:GLU:HB2	2.52	0.44
1:B:274:HIS:CD2	1:B:286:ALA:H	2.28	0.44
1:B:459:GLN:NE2	1:B:477:GLY:HA2	2.33	0.44
1:A:215:SER:O	1:A:268:ARG:NH2	2.52	0.43
1:B:369:ILE:HG23	1:B:373:ALA:HB2	2.01	0.43
1:A:304:LYS:HB2	1:A:358:ASN:HB3	2.01	0.42
1:B:277[A]:CYS:SG	2:B:601:UTP:C4'	3.08	0.42
1:A:104:SER:O	1:A:108:SER:HB2	2.20	0.42
1:A:236:MET:O	1:A:387:CYS:HB2	2.20	0.42
1:A:138:GLN:HE21	1:A:195:PRO:HG3	1.84	0.41
1:A:129:VAL:O	1:A:187:PRO:HD2	2.21	0.41
1:A:435:PRO:HB2	1:A:437:LYS:HD2	2.02	0.41
1:A:246:PRO:HB2	2:A:601[B]:UTP:O4	2.21	0.41
1:B:456:SER:O	1:B:460:ARG:HG3	2.21	0.41
1:A:266:ARG:HH22	1:A:367:GLU:HG2	1.86	0.40
1:A:458:GLY:HA3	1:A:478:VAL:O	2.21	0.40
1:A:302:ALA:HB3	1:A:360:TYR:HB3	2.04	0.40
1:B:446:ASP:CB	1:B:447:PRO:CD	2.96	0.40
1:B:154:GLY:O	1:B:451:LYS:HE3	2.21	0.40

There are no symmetry-related clashes.

CONFIDENTIAL VALIDATION REPORT

5.3 Torsion angles (i)

5.3.1 Protein backbone (i)

In the following table, the Percentiles column shows the percent Ramachandran outliers of the chain as a percentile score with respect to all X-ray entries followed by that with respect to entries of similar resolution.

The Analysed column shows the number of residues for which the backbone conformation was analysed, and the total number of residues.

Mol	Chain	Analysed	Favoured	Allowed	Outliers	Percentiles	
1	A	452/509 (89%)	435 (96%)	17 (4%)	0	100	100
1	B	461/509 (91%)	447 (97%)	14 (3%)	0	100	100
All	All	913/1018 (90%)	882 (97%)	31 (3%)	0	100	100

There are no Ramachandran outliers to report.

5.3.2 Protein sidechains (i)

In the following table, the Percentiles column shows the percent sidechain outliers of the chain as a percentile score with respect to all X-ray entries followed by that with respect to entries of similar resolution.

The Analysed column shows the number of residues for which the sidechain conformation was analysed, and the total number of residues.

Mol	Chain	Analysed	Rotameric	Outliers	Percentiles	
1	A	391/431 (91%)	382 (98%)	9 (2%)	53	73
1	B	398/431 (92%)	381 (96%)	17 (4%)	32	49
All	All	789/862 (92%)	763 (97%)	26 (3%)	41	61

All (26) residues with a non-rotameric sidechain are listed below:

Mol	Chain	Res	Type
1	A	30	GLU
1	A	37	LYS
1	A	73	ARG
1	A	111	LYS
1	A	208	LYS
1	A	319	LEU
1	A	320	GLN
1	A	384	LYS

Continued on next page...

CONFIDENTIAL VALIDATION REPORT

Continued from previous page...

Mol	Chain	Res	Type
1	A	507	LYS
1	B	94	LEU
1	B	97	LEU
1	B	100	ILE
1	B	111	LYS
1	B	172	LEU
1	B	233	LYS
1	B	275	THR
1	B	307	ARG
1	B	309	ARG
1	B	337	THR
1	B	341	LYS
1	B	347	ASP
1	B	349	LEU
1	B	358	ASN
1	B	364	LYS
1	B	388	ILE
1	B	507	LYS

Some sidechains can be flipped to improve hydrogen bonding and reduce clashes. All (19) such sidechains are listed below:

Mol	Chain	Res	Type
1	A	158	HIS
1	A	163	GLN
1	A	173	GLN
1	A	230	ASN
1	A	274	HIS
1	A	322	ASN
1	A	359	HIS
1	A	378	HIS
1	A	379	HIS
1	B	138	GLN
1	B	173	GLN
1	B	177	GLN
1	B	274	HIS
1	B	320	GLN
1	B	358	ASN
1	B	359	HIS
1	B	378	HIS
1	B	457	GLN
1	B	459	GLN

CONFIDENTIAL VALIDATION REPORT

5.3.3 RNA (i)

There are no RNA molecules in this entry.

5.4 Non-standard residues in protein, DNA, RNA chains (i)

There are no non-standard protein/DNA/RNA residues in this entry.

5.5 Carbohydrates (i)

There are no carbohydrates in this entry.

5.6 Ligand geometry (i)

3 ligands are modelled in this entry.

In the following table, the Counts columns list the number of bonds (or angles) for which Mogul statistics could be retrieved, the number of bonds (or angles) that are observed in the model and the number of bonds (or angles) that are defined in the Chemical Component Dictionary. The Link column lists molecule types, if any, to which the group is linked. The Z score for a bond length (or angle) is the number of standard deviations the observed value is removed from the expected value. A bond length (or angle) with $|Z| > 2$ is considered an outlier worth inspection. RMSZ is the root-mean-square of all Z scores of the bond lengths (or angles).

Mol	Type	Chain	Res	Link	Bond lengths			Bond angles		
					Counts	RMSZ	$\#\|Z\| > 2$	Counts	RMSZ	$\#\|Z\| > 2$
2	UTP	A	601[A]	-	26,30,30	2.26	6 (23%)	32,47,47	2.50	6 (18%)
2	UTP	A	601[B]	-	26,30,30	2.27	8 (30%)	32,47,47	2.36	5 (15%)
2	UTP	B	601	-	26,30,30	2.19	6 (23%)	32,47,47	2.39	4 (12%)

In the following table, the Chirals column lists the number of chiral outliers, the number of chiral centers analysed, the number of these observed in the model and the number defined in the Chemical Component Dictionary. Similar counts are reported in the Torsion and Rings columns. '-' means no outliers of that kind were identified.

Mol	Type	Chain	Res	Link	Chirals	Torsions	Rings
2	UTP	A	601[A]	-	-	0/22/38/38	0/2/2/2
2	UTP	A	601[B]	-	-	0/22/38/38	0/2/2/2
2	UTP	B	601	-	-	0/22/38/38	0/2/2/2

All (20) bond length outliers are listed below:

CONFIDENTIAL VALIDATION REPORT

Mol	Chain	Res	Type	Atoms	Z	Observed(Å)	Ideal(Å)
2	A	601[B]	UTP	C6-C5	-6.88	1.39	1.52
2	B	601	UTP	C6-C5	-6.77	1.39	1.52
2	A	601[A]	UTP	C6-C5	-6.51	1.40	1.52
2	A	601[A]	UTP	C5-C4	-4.40	1.40	1.50
2	B	601	UTP	C5-C4	-4.37	1.40	1.50
2	A	601[B]	UTP	C5-C4	-4.34	1.40	1.50
2	A	601[A]	UTP	C6-N1	-3.87	1.42	1.47
2	A	601[B]	UTP	C6-N1	-3.81	1.42	1.47
2	B	601	UTP	C6-N1	-3.39	1.42	1.47
2	B	601	UTP	C4-N3	2.08	1.40	1.37
2	A	601[B]	UTP	O4'-C1'	2.10	1.47	1.42
2	A	601[A]	UTP	C4-N3	2.13	1.40	1.37
2	B	601	UTP	O4'-C1'	2.28	1.47	1.42
2	A	601[B]	UTP	PG-O1G	2.28	1.64	1.54
2	A	601[B]	UTP	C4-N3	2.43	1.41	1.37
2	A	601[B]	UTP	PG-O2G	3.21	1.61	1.50
2	A	601[A]	UTP	PG-O2G	3.64	1.62	1.50
2	A	601[B]	UTP	C2-N1	3.78	1.41	1.35
2	B	601	UTP	C2-N1	4.22	1.41	1.35
2	A	601[A]	UTP	C2-N1	4.37	1.42	1.35

All (15) bond angle outliers are listed below:

Mol	Chain	Res	Type	Atoms	Z	Observed(^{o})	Ideal(^{o})
2	B	601	UTP	C4-N3-C2	-6.04	120.63	125.81
2	A	601[A]	UTP	C4-N3-C2	-5.92	120.74	125.81
2	A	601[B]	UTP	C4-N3-C2	-5.52	121.08	125.81
2	A	601[A]	UTP	PB-O3B-PG	-3.50	120.86	132.63
2	A	601[B]	UTP	PB-O3A-PA	-3.26	121.66	132.63
2	A	601[B]	UTP	PB-O3B-PG	-3.18	121.94	132.63
2	B	601	UTP	PB-O3A-PA	-2.96	122.69	132.63
2	A	601[A]	UTP	PB-O3A-PA	-2.21	125.20	132.63
2	A	601[A]	UTP	C5-C4-N3	3.05	119.64	116.73
2	A	601[A]	UTP	N3-C2-N1	3.39	120.07	116.73
2	A	601[B]	UTP	C5-C4-N3	3.54	120.11	116.73
2	B	601	UTP	C5-C4-N3	4.38	120.90	116.73
2	B	601	UTP	C5-C6-N1	9.60	120.61	110.71
2	A	601[B]	UTP	C5-C6-N1	9.64	120.65	110.71
2	A	601[A]	UTP	C5-C6-N1	10.07	121.10	110.71

There are no chirality outliers.

There are no torsion outliers.

CONFIDENTIAL VALIDATION REPORT

There are no ring outliers.

3 monomers are involved in 10 short contacts:

Mol	Chain	Res	Type	Clashes	Symm-Clashes
2	A	601[A]	UTP	1	0
2	A	601[B]	UTP	3	0
2	B	601	UTP	6	0

5.7 Other polymers (i)

There are no such residues in this entry.

5.8 Polymer linkage issues (i)

There are no chain breaks in this entry.

CONFIDENTIAL VALIDATION REPORT

6 Fit of model and data (i)

6.1 Protein, DNA and RNA chains (i)

In the following table, the column labelled '#RSRZ> 2' contains the number (and percentage) of RSRZ outliers, followed by percent RSRZ outliers for the chain as percentile scores relative to all X-ray entries and entries of similar resolution. The OWAB column contains the minimum, median, 95th percentile and maximum values of the occupancy-weighted average B-factor per residue. The column labelled 'Q< 0.9' lists the number of (and percentage) of residues with an average occupancy less than 0.9.

Mol	Chain	Analysed	<RSRZ>	#RSRZ>2			OWAB(Å^2)	Q<0.9
1	A	460/509 (90%)	-0.15	4 (0%)	84	82	22, 36, 51, 63	0
1	B	468/509 (91%)	-0.25	7 (1%)	73	71	17, 31, 52, 75	0
All	All	928/1018 (91%)	-0.20	11 (1%)	79	77	17, 33, 52, 75	0

All (11) RSRZ outliers are listed below:

Mol	Chain	Res	Type	RSRZ
1	B	395	PHE	3.0
1	B	396	PHE	3.0
1	B	397	LYS	2.8
1	B	470	ILE	2.7
1	A	84	PRO	2.7
1	B	347	ASP	2.6
1	A	472	GLU	2.4
1	B	314	SER	2.3
1	B	390	GLU	2.3
1	A	384	LYS	2.2
1	A	298	GLN	2.1

6.2 Non-standard residues in protein, DNA, RNA chains (i)

There are no non-standard protein/DNA/RNA residues in this entry.

6.3 Carbohydrates (i)

There are no carbohydrates in this entry.

CONFIDENTIAL VALIDATION REPORT

6.4 Ligands (i)

In the following table, the Atoms column lists the number of modelled atoms in the group and the number defined in the chemical component dictionary. The B-factors column lists the minimum, median, 95th percentile and maximum values of B factors of atoms in the group. The column labelled 'Q< 0.9' lists the number of atoms with occupancy less than 0.9.

Mol	Type	Chain	Res	Atoms	RSCC	RSR	B-factors(Å^2)	Q<0.9
2	UTP	A	601[B]	29/29	0.88	0.21	22,27,37,38	29
2	UTP	A	601[A]	29/29	0.88	0.21	44,45,55,55	29
2	UTP	B	601	29/29	0.90	0.17	45,51,53,53	0

6.5 Other polymers (i)

There are no such residues in this entry.

CONFIDENTIAL VALIDATION REPORT
