# Supplementary material for: A mechanism-inspired UDP-N-acetylglucosamine pyrophosphorylase inhibitor
Source: RSC Chem Biol. 2020 Mar 24;1(1):13–25. doi: 10.1039/c9cb00017h (PMC8386105; doi:10.1039/c9cb00017h)
Supplement: CB-001-C9CB00017H-s006 [file CB-001-C9CB00017H-s006.pdf]

Full wwPDB X-ray Structure Validation Report (i)

Dec 6, 2019 – 10:45 am GMT

PDB ID	:	6TN3
Title	:	Crystal Structure of Aspergillus fumigatus UDP-N-acetylglucosamine pyrophosphorylase in complex with GlcNAc-1P
Deposited on	:	2019-12-05
Resolution	:	2.28 Å(reported)

This is a Full wwPDB X-ray Structure Validation Report.

This report is produced by the wwPDB biocuration pipeline after annotation of the structure.

We welcome your comments at *validation@mail.wwpdb.org*
A user guide is available at
https://www.wwpdb.org/validation/2017/XrayValidationReportHelp
with specific help available everywhere you see the (i) symbol.

CONFIDENTIAL VALIDATION REPORT

The following versions of software and data (see references (i)) were used in the production of this report:

MolProbity	:	4.02b-467
Mogul	:	1.8.0 (224370), CSD as540be (2019)
Xtriage (Phenix)	:	1.13
EDS	:	2.6.1
buster-report	:	1.1.7 (2018)
Percentile statistics	:	20171227.v01 (using entries in the PDB archive December 27th 2017)
Refmac	:	5.8.0158
CCP4	:	7.0.044 (Gargrove)
Ideal geometry (proteins)	:	Engh & Huber (2001)
Ideal geometry (DNA, RNA)	:	Parkinson et al. (1996)
Validation Pipeline (wwPDB-VP)	:	2.6.1

1 Overall quality at a glance (i)

The following experimental techniques were used to determine the structure:
X-RAY DIFFRACTION

The reported resolution of this entry is 2.28 Å.

Percentile scores (ranging between 0-100) for global validation metrics of the entry are shown in the following graphic. The table shows the number of entries on which the scores are based.

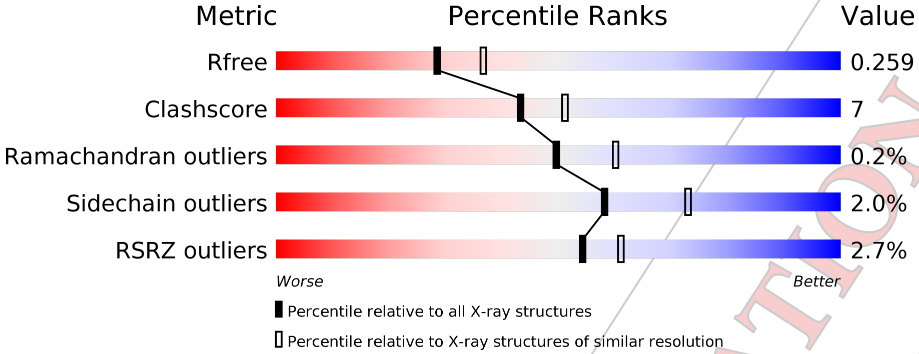

Metric	Whole archive (#Entries)	Similar resolution (#Entries, resolution range(Å))
R_{free}	111664	6121 (2.30-2.26)
Clashscore	122126	6842 (2.30-2.26)
Ramachandran outliers	120053	6755 (2.30-2.26)
Sidechain outliers	120020	6755 (2.30-2.26)
RSRZ outliers	108989	5992 (2.30-2.26)

The table below summarises the geometric issues observed across the polymeric chains and their fit to the electron density. The red, orange, yellow and green segments on the lower bar indicate the fraction of residues that contain outliers for >=3, 2, 1 and 0 types of geometric quality criteria respectively. A grey segment represents the fraction of residues that are not modelled. The numeric value for each fraction is indicated below the corresponding segment, with a dot representing fractions <=5% The upper red bar (where present) indicates the fraction of residues that have poor fit to the electron density. The numeric value is given above the bar.

Mol	Chain	Length	Quality of chain
1	A	509	4% / 71% 15% • 13%
1	B	509	% / 76% 14% • 9%

The following table lists non-polymeric compounds, carbohydrate monomers and non-standard residues in protein, DNA, RNA chains that are outliers for geometric or electron-density-fit criteria:

CONFIDENTIAL VALIDATION REPORT

Mol	Type	Chain	Res	Chirality	Geometry	Clashes	Electron density
3	GN1	B	602	X	-	-	-

CONFIDENTIAL VALIDATION REPORT

2 Entry composition (i)

There are 4 unique types of molecules in this entry. The entry contains 7374 atoms, of which 0 are hydrogens and 0 are deuteriums.

In the tables below, the ZeroOcc column contains the number of atoms modelled with zero occupancy, the AltConf column contains the number of residues with at least one atom in alternate conformation and the Trace column contains the number of residues modelled with at most 2 atoms.

- Molecule 1 is a protein called UDP-N-acetylglucosamine pyrophosphorylase.

Mol	Chain	Residues	Atoms					ZeroOcc	AltConf	Trace
1	A	442	Total 3482	C 2228	N 594	O 647	S 13	0	2	0
1	B	463	Total 3637	C 2323	N 622	O 680	S 12	0	0	0

- Molecule 2 is PHOSPHATE ION (three-letter code: PO4) (formula: O_4P).

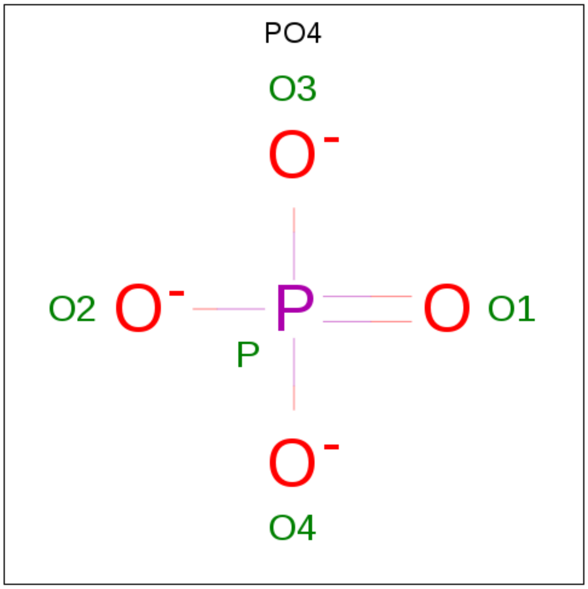

Mol	Chain	Residues	Atoms			ZeroOcc	AltConf
2	B	1	Total 5	O 4	P 1	0	0

- Molecule 3 is 2-(ACETYLAMINO)-2-DEOXY-1-O-PHOSPHONO-ALPHA-D-GLUCOPYRANOSE (three-letter code: GN1) (formula: $C_8H_{16}NO_9P$) (labeled as "Ligand of Interest" by author).

CONFIDENTIAL VALIDATION REPORT

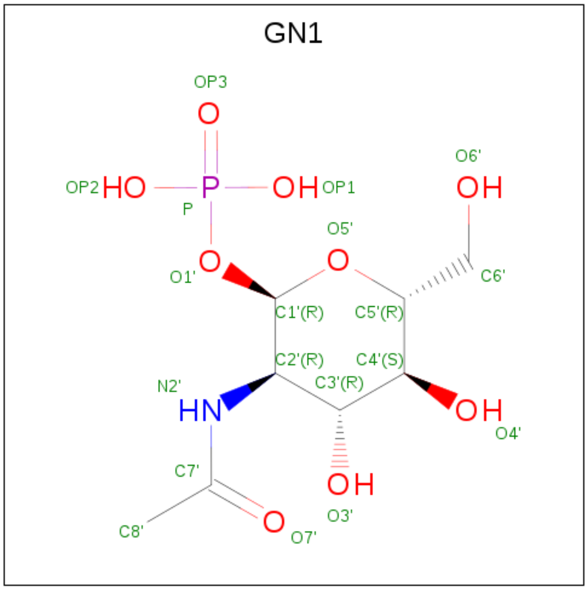

Mol	Chain	Residues	Atoms					ZeroOcc	AltConf
			Total	C	N	O	P		
3	B	1	19	8	1	9	1	0	0

- Molecule 4 is water.

Mol	Chain	Residues	Atoms		ZeroOcc	AltConf
			Total	O		
4	A	101	101	101	0	0
4	B	130	130	130	0	0

CONFIDENTIAL VALIDATION REPORT

3 Residue-property plots ⓘ

These plots are drawn for all protein, RNA and DNA chains in the entry. The first graphic for a chain summarises the proportions of the various outlier classes displayed in the second graphic. The second graphic shows the sequence view annotated by issues in geometry and electron density. Residues are color-coded according to the number of geometric quality criteria for which they contain at least one outlier: green = 0, yellow = 1, orange = 2 and red = 3 or more. A red dot above a residue indicates a poor fit to the electron density (RSRZ > 2). Stretches of 2 or more consecutive residues without any outlier are shown as a green connector. Residues present in the sample, but not in the model, are shown in grey.

- Molecule 1: UDP-N-acetylglucosamine pyrophosphorylase

Chain A:

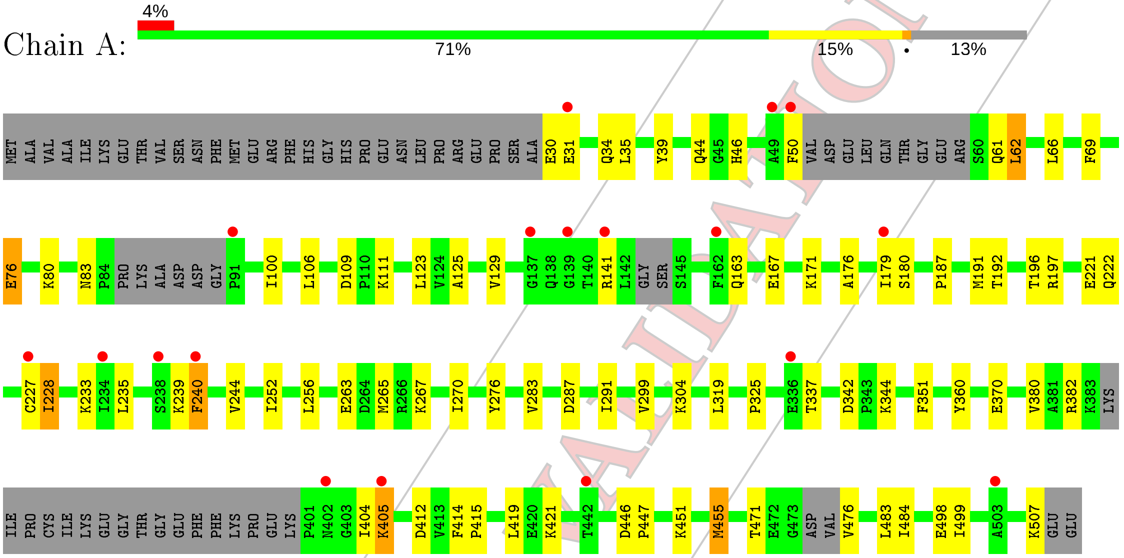

- Molecule 1: UDP-N-acetylglucosamine pyrophosphorylase

Chain B:

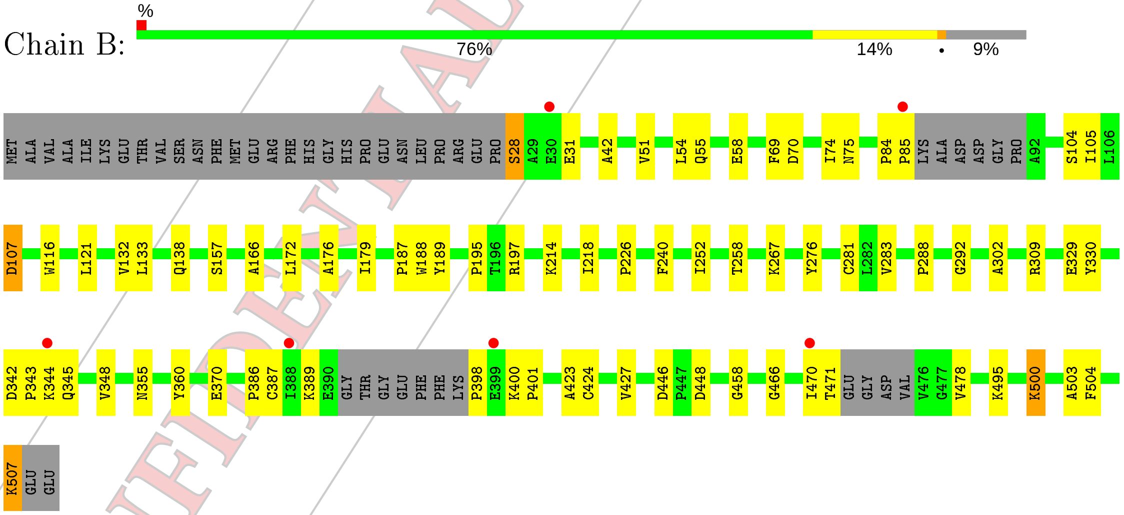

CONFIDENTIAL VALIDATION REPORT

4 Data and refinement statistics (i)

Property	Value	Source
Space group	P 21 21 21	Depositor
Cell constants<br>a, b, c, α, β, γ	55.74Å 138.14Å 145.13Å<br>90.00° 90.00° 90.00°	Depositor
Resolution (Å)	19.93 – 2.28<br>19.92 – 2.28	Depositor<br>EDS
% Data completeness<br>(in resolution range)	95.5 (19.93-2.28)<br>95.5 (19.92-2.28)	Depositor<br>EDS
R_{merge}	0.12	Depositor
R_{sym}	(Not available)	Depositor
$< I/\sigma(I) >$ [1]	2.14 (at 2.28Å)	Xtriage
Refinement program	PHENIX 1.10.1_2155	Depositor
R, R_{free}	0.205 , 0.259<br>0.205 , 0.259	Depositor<br>DCC
R_{free} test set	2507 reflections (5.07%)	wwPDB-VP
Wilson B-factor (Å^2)	43.7	Xtriage
Anisotropy	0.276	Xtriage
Bulk solvent k_{sol}(e/Å^3), B_{sol}(Å^2)	0.30 , 40.9	EDS
L-test for twinning[2]	$< \|L\| >= 0.48$, $< L^2 >= 0.31$	Xtriage
Estimated twinning fraction	0.006 for -h,l,k	Xtriage
F_o,F_c correlation	0.94	EDS
Total number of atoms	7374	wwPDB-VP
Average B, all atoms (Å^2)	48.0	wwPDB-VP

Xtriage's analysis on translational NCS is as follows: *The largest off-origin peak in the Patterson function is 3.55% of the height of the origin peak. No significant pseudotranslation is detected.*

[1]Intensities estimated from amplitudes.

[2]Theoretical values of $< |L| >$, $< L^2 >$ for acentric reflections are 0.5, 0.333 respectively for untwinned datasets, and 0.375, 0.2 for perfectly twinned datasets.

CONFIDENTIAL VALIDATION REPORT

5 Model quality ⓘ

5.1 Standard geometry ⓘ

Bond lengths and bond angles in the following residue types are not validated in this section: PO4, GN1

The Z score for a bond length (or angle) is the number of standard deviations the observed value is removed from the expected value. A bond length (or angle) with $|Z| > 5$ is considered an outlier worth inspection. RMSZ is the root-mean-square of all Z scores of the bond lengths (or angles).

Mol	Chain	Bond lengths		Bond angles	
		RMSZ	#$|Z|$ >5	RMSZ	#$|Z|$ >5
1	A	0.44	0/3560	0.60	3/4796 (0.1%)
1	B	0.46	1/3713 (0.0%)	0.58	0/5006
All	All	0.45	1/7273 (0.0%)	0.59	3/9802 (0.0%)

Chiral center outliers are detected by calculating the chiral volume of a chiral center and verifying if the center is modelled as a planar moiety or with the opposite hand.A planarity outlier is detected by checking planarity of atoms in a peptide group, atoms in a mainchain group or atoms of a sidechain that are expected to be planar.

Mol	Chain	#Chirality outliers	#Planarity outliers
1	B	0	1

All (1) bond length outliers are listed below:

Mol	Chain	Res	Type	Atoms	Z	Observed(Å)	Ideal(Å)
1	B	424	CYS	CB-SG	-5.79	1.72	1.81

All (3) bond angle outliers are listed below:

Mol	Chain	Res	Type	Atoms	Z	Observed(^{o})	Ideal(^{o})
1	A	62	LEU	CB-CG-CD1	-8.21	97.05	111.00
1	A	405	LYS	CD-CE-NZ	-6.64	96.44	111.70
1	A	405	LYS	CB-CG-CD	-5.48	97.35	111.60

There are no chirality outliers.

All (1) planarity outliers are listed below:

Mol	Chain	Res	Type	Group
1	B	389	LYS	Peptide

CONFIDENTIAL VALIDATION REPORT

5.2 Too-close contacts (i)

In the following table, the Non-H and H(model) columns list the number of non-hydrogen atoms and hydrogen atoms in the chain respectively. The H(added) column lists the number of hydrogen atoms added and optimized by MolProbity. The Clashes column lists the number of clashes within the asymmetric unit, whereas Symm-Clashes lists symmetry related clashes.

Mol	Chain	Non-H	H(model)	H(added)	Clashes	Symm-Clashes
1	A	3482	0	3496	56	0
1	B	3637	0	3655	48	0
2	B	5	0	0	0	0
3	B	19	0	14	2	0
4	A	101	0	0	0	0
4	B	130	0	0	4	0
All	All	7374	0	7165	104	0

The all-atom clashscore is defined as the number of clashes found per 1000 atoms (including hydrogen atoms). The all-atom clashscore for this structure is 7.

All (104) close contacts within the same asymmetric unit are listed below, sorted by their clash magnitude.

Atom-1	Atom-2	Interatomic distance (Å)	Clash overlap (Å)
1:A:50:PHE:HA	1:A:239:LYS:HE2	1.35	1.08
1:A:30:GLU:HG2	1:A:31:GLU:H	1.23	1.03
1:B:345:GLN:HB2	1:B:348:VAL:HG12	1.59	0.84
1:A:50:PHE:HA	1:A:239:LYS:CE	2.10	0.80
1:B:344:LYS:NZ	4:B:701:HOH:O	2.17	0.76
1:B:138:GLN:HE22	1:B:195:PRO:HG2	1.49	0.76
1:B:104:SER:HB3	1:B:107:ASP:HB2	1.69	0.74
1:A:319:LEU:HD21	1:A:351:PHE:HD1	1.54	0.72
1:A:62:LEU:HD11	1:A:240:PHE:HA	1.74	0.70
1:A:30:GLU:HG2	1:A:31:GLU:N	2.04	0.69
1:A:125:ALA:HB1	1:A:180:SER:HB3	1.76	0.68
1:B:345:GLN:HB2	1:B:348:VAL:CG1	2.25	0.67
1:A:484:ILE:HD11	1:A:507:LYS:HA	1.77	0.67
1:A:141:ARG:NH1	1:A:141:ARG:O	2.29	0.66
1:B:55:GLN:HB2	1:B:58:GLU:HG3	1.78	0.64
1:A:30:GLU:CG	1:A:31:GLU:H	2.08	0.63
1:A:100:ILE:H	1:A:100:ILE:HD12	1.66	0.61
1:A:325:PRO:HD3	1:A:419:LEU:HD13	1.84	0.60
1:A:227:CYS:CB	1:A:405:LYS:HZ1	2.15	0.58
1:B:157:SER:HB3	1:B:504:PHE:CZ	2.38	0.58
1:A:76:GLU:OE1	1:A:80:LYS:NZ	2.24	0.58

Continued on next page...

CONFIDENTIAL VALIDATION REPORT

Continued from previous page...

Atom-1	Atom-2	Interatomic distance (Å)	Clash overlap (Å)
1:B:355:ASN:ND2	3:B:602:GN1:H3'	2.19	0.57
1:B:138:GLN:NE2	1:B:195:PRO:HG2	2.19	0.56
1:B:189:TYR:CE1	1:B:218:ILE:HD13	2.41	0.56
1:B:466:GLY:HA3	1:B:495:LYS:HG3	1.88	0.55
1:A:446:ASP:HB2	1:A:447:PRO:HD2	1.87	0.54
1:A:109:ASP:OD1	1:A:111:LYS:HG2	2.06	0.54
1:A:228:ILE:HA	1:A:233:LYS:O	2.07	0.54
1:B:214:LYS:HG3	4:B:777:HOH:O	2.08	0.53
1:B:28:SER:HB3	1:B:31:GLU:OE1	2.07	0.53
1:B:281:CYS:HB3	4:B:807:HOH:O	2.08	0.53
1:A:227:CYS:O	1:A:228:ILE:HG12	2.08	0.53
1:B:343:PRO:HB2	1:B:344:LYS:HE2	1.91	0.53
1:B:500:LYS:O	1:B:503:ALA:HB2	2.10	0.51
1:B:132:VAL:HG11	1:B:252:ILE:HD11	1.93	0.51
1:A:287:ASP:OD2	1:A:304:LYS:NZ	2.36	0.50
1:A:69:PHE:HZ	1:A:244:VAL:HG11	1.77	0.50
1:A:39:TYR:OH	1:A:69:PHE:HB2	2.13	0.49
1:A:380:VAL:HG23	1:A:404:ILE:HG23	1.95	0.49
1:B:386:PRO:HA	1:B:398:PRO:HG3	1.95	0.48
1:B:51:VAL:HA	1:B:54:LEU:HD12	1.95	0.48
1:A:342:ASP:OD2	1:A:344:LYS:N	2.46	0.48
1:B:345:GLN:O	1:B:348:VAL:HG12	2.14	0.48
1:B:69:PHE:CE1	1:B:226:PRO:HD3	2.49	0.48
1:B:54:LEU:HB3	1:B:58:GLU:HB2	1.96	0.47
1:B:446:ASP:OD1	1:B:448:ASP:N	2.47	0.47
1:A:62:LEU:HD11	1:A:240:PHE:CA	2.44	0.47
1:A:263:GLU:OE2	1:A:267:LYS:NZ	2.47	0.47
1:A:197:ARG:HD2	1:A:221:GLU:CG	2.44	0.47
1:B:166:ALA:HB2	1:B:188:TRP:CZ3	2.50	0.47
1:B:458:GLY:HA3	1:B:478:VAL:O	2.16	0.46
1:A:171:LYS:NZ	1:A:484:ILE:O	2.48	0.46
1:B:84:PRO:HA	1:B:85:PRO:HD3	1.85	0.46
1:A:35:LEU:HD21	1:A:66:LEU:HB2	1.98	0.46
1:A:227:CYS:HA	1:A:405:LYS:NZ	2.30	0.46
1:A:163:GLN:O	1:A:167:GLU:HG3	2.15	0.46
1:A:106:LEU:HD12	1:A:106:LEU:H	1.79	0.45
1:B:42:ALA:HB1	1:B:75:ASN:ND2	2.32	0.45
1:A:276:TYR:CE2	1:A:283:VAL:HG11	2.51	0.45
1:A:451:LYS:O	1:A:455:MET:HG3	2.15	0.45
1:A:299:VAL:HB	1:A:421:LYS:O	2.17	0.45
1:A:319:LEU:HD23	1:A:319:LEU:HA	1.65	0.45

Continued on next page...

CONFIDENTIAL VALIDATION REPORT

Continued from previous page...

Atom-1	Atom-2	Interatomic distance (Å)	Clash overlap (Å)
1:A:370:GLU:N	1:A:370:GLU:OE2	2.39	0.45
1:B:276:TYR:CD2	1:B:283:VAL:HG21	2.52	0.45
1:A:176:ALA:O	1:A:179:ILE:HG12	2.17	0.45
1:B:105:ILE:HG13	1:B:427:VAL:HG12	1.99	0.45
1:B:342:ASP:HB3	1:B:348:VAL:HG13	1.98	0.44
1:B:116:TRP:O	1:B:292:GLY:HA3	2.18	0.44
1:A:414:PHE:HB2	1:A:415:PRO:HD3	2.00	0.44
1:A:191:MET:SD	1:A:222:GLN:HB3	2.58	0.44
1:A:192:THR:HB	1:A:196:THR:OG1	2.18	0.43
1:A:227:CYS:HB2	1:A:235:LEU:HB2	2.00	0.43
1:B:70:ASP:O	1:B:74:ILE:HG12	2.18	0.43
1:A:31:GLU:HA	1:A:34:GLN:HG3	2.00	0.43
1:A:404:ILE:O	1:A:405:LYS:HD2	2.19	0.43
1:A:471:THR:HG21	1:A:476:VAL:HG23	1.99	0.43
1:A:197:ARG:HD2	1:A:221:GLU:HG2	2.00	0.43
1:B:187:PRO:HB2	1:B:189:TYR:CE2	2.54	0.42
1:B:133:LEU:HD13	1:B:276:TYR:CZ	2.54	0.42
1:A:498:GLU:O	1:A:499:ILE:HG13	2.19	0.42
1:B:344:LYS:HD3	1:B:344:LYS:HA	1.70	0.42
1:A:61:GLN:H	1:A:240:PHE:HE1	1.68	0.42
1:A:123:LEU:HD12	1:A:291:ILE:HG22	2.02	0.42
1:A:360:TYR:CD1	1:A:360:TYR:C	2.92	0.42
1:B:302:ALA:HA	1:B:423:ALA:O	2.20	0.42
1:B:507:LYS:HE3	1:B:507:LYS:HB3	1.79	0.41
1:B:302:ALA:HB3	1:B:360:TYR:HB3	2.02	0.41
1:B:176:ALA:O	1:B:179:ILE:HG13	2.20	0.41
1:B:370:GLU:HB3	4:B:825:HOH:O	2.20	0.41
1:B:330:TYR:OH	3:B:602:GN1:OP3	2.26	0.41
1:B:400:LYS:HD3	1:B:401:PRO:HD2	2.02	0.41
1:A:319:LEU:HD21	1:A:351:PHE:CD1	2.44	0.41
1:A:265:MET:HB3	1:A:270:ILE:HB	2.03	0.41
1:B:470:ILE:O	1:B:471:THR:HG22	2.20	0.41
1:A:483:LEU:HB3	1:A:507:LYS:HD3	2.02	0.40
1:B:54:LEU:HD22	1:B:240:PHE:HB3	2.03	0.40
1:A:252:ILE:O	1:A:256:LEU:HG	2.21	0.40
1:A:44:GLN:OE1	1:A:46:HIS:HE1	2.04	0.40
1:B:172:LEU:HD11	1:B:288:PRO:HG3	2.03	0.40
1:B:329:GLU:OE1	1:B:329:GLU:HA	2.21	0.40
1:B:500:LYS:HD2	1:B:500:LYS:O	2.20	0.40
1:A:412:ASP:O	1:A:415:PRO:HD2	2.21	0.40
1:A:129:VAL:O	1:A:187:PRO:HD2	2.21	0.40

Continued on next page...

CONFIDENTIAL VALIDATION REPORT

Continued from previous page...

Atom-1	Atom-2	Interatomic distance (Å)	Clash overlap (Å)
1:B:105:ILE:CG1	1:B:427:VAL:HG12	2.52	0.40

There are no symmetry-related clashes.

5.3 Torsion angles (i)

5.3.1 Protein backbone (i)

In the following table, the Percentiles column shows the percent Ramachandran outliers of the chain as a percentile score with respect to all X-ray entries followed by that with respect to entries of similar resolution.

The Analysed column shows the number of residues for which the backbone conformation was analysed, and the total number of residues.

Mol	Chain	Analysed	Favoured	Allowed	Outliers	Percentiles	
1	A	432/509 (85%)	416 (96%)	15 (4%)	1 (0%)	49	60
1	B	455/509 (89%)	443 (97%)	11 (2%)	1 (0%)	49	60
All	All	887/1018 (87%)	859 (97%)	26 (3%)	2 (0%)	49	60

All (2) Ramachandran outliers are listed below:

Mol	Chain	Res	Type
1	A	228	ILE
1	B	387	CYS

5.3.2 Protein sidechains (i)

In the following table, the Percentiles column shows the percent sidechain outliers of the chain as a percentile score with respect to all X-ray entries followed by that with respect to entries of similar resolution.

The Analysed column shows the number of residues for which the sidechain conformation was analysed, and the total number of residues.

Mol	Chain	Analysed	Rotameric	Outliers	Percentiles	
1	A	376/431 (87%)	370 (98%)	6 (2%)	65	79
1	B	393/431 (91%)	384 (98%)	9 (2%)	53	68
All	All	769/862 (89%)	754 (98%)	15 (2%)	58	73

CONFIDENTIAL VALIDATION REPORT

All (15) residues with a non-rotameric sidechain are listed below:

Mol	Chain	Res	Type
1	A	76	GLU
1	A	83	ASN
1	A	240	PHE
1	A	337	THR
1	A	382	ARG
1	A	455	MET
1	B	28	SER
1	B	107	ASP
1	B	121	LEU
1	B	197	ARG
1	B	258	THR
1	B	267	LYS
1	B	309	ARG
1	B	500	LYS
1	B	507	LYS

Some sidechains can be flipped to improve hydrogen bonding and reduce clashes. All (2) such sidechains are listed below:

Mol	Chain	Res	Type
1	A	83	ASN
1	B	138	GLN

5.3.3 RNA (i)

There are no RNA molecules in this entry.

5.4 Non-standard residues in protein, DNA, RNA chains (i)

There are no non-standard protein/DNA/RNA residues in this entry.

5.5 Carbohydrates (i)

There are no carbohydrates in this entry.

5.6 Ligand geometry (i)

2 ligands are modelled in this entry.

CONFIDENTIAL VALIDATION REPORT

In the following table, the Counts columns list the number of bonds (or angles) for which Mogul statistics could be retrieved, the number of bonds (or angles) that are observed in the model and the number of bonds (or angles) that are defined in the Chemical Component Dictionary. The Link column lists molecule types, if any, to which the group is linked. The Z score for a bond length (or angle) is the number of standard deviations the observed value is removed from the expected value. A bond length (or angle) with $|Z| > 2$ is considered an outlier worth inspection. RMSZ is the root-mean-square of all Z scores of the bond lengths (or angles).

Mol	Type	Chain	Res	Link	Bond lengths			Bond angles		
					Counts	RMSZ	$\#\|Z\| > 2$	Counts	RMSZ	$\#\|Z\| > 2$
2	PO4	B	601	-	4,4,4	0.73	0	6,6,6	0.51	0
3	GN1	B	602	-	18,19,19	3.06	5 (27%)	27,28,28	2.16	11 (40%)

In the following table, the Chirals column lists the number of chiral outliers, the number of chiral centers analysed, the number of these observed in the model and the number defined in the Chemical Component Dictionary. Similar counts are reported in the Torsion and Rings columns. '-' means no outliers of that kind were identified.

Mol	Type	Chain	Res	Link	Chirals	Torsions	Rings
3	GN1	B	602	-	1/1/7/8	1/10/31/31	0/1/1/1

All (5) bond length outliers are listed below:

Mol	Chain	Res	Type	Atoms	Z	Observed(Å)	Ideal(Å)
3	B	602	GN1	P-O1'	10.03	1.78	1.59
3	B	602	GN1	C1'-C2'	5.27	1.61	1.53
3	B	602	GN1	C4'-C3'	3.17	1.60	1.52
3	B	602	GN1	C4'-C5'	2.51	1.58	1.53
3	B	602	GN1	C6'-C5'	2.29	1.59	1.51

All (11) bond angle outliers are listed below:

Mol	Chain	Res	Type	Atoms	Z	Observed(^{o})	Ideal(^{o})
3	B	602	GN1	C6'-C5'-C4'	-4.51	102.40	113.00
3	B	602	GN1	O5'-C1'-O1'	3.69	116.19	111.36
3	B	602	GN1	C3'-C4'-C5'	3.60	116.70	110.23
3	B	602	GN1	O1'-C1'-C2'	3.23	114.25	108.40
3	B	602	GN1	C1'-O5'-C5'	3.07	119.74	113.70
3	B	602	GN1	C4'-C3'-C2'	2.91	114.67	110.35
3	B	602	GN1	OP2-P-OP1	2.85	118.64	107.57
3	B	602	GN1	O5'-C1'-C2'	-2.52	105.58	110.58
3	B	602	GN1	O1'-P-OP3	-2.34	100.36	109.39
3	B	602	GN1	O3'-C3'-C4'	2.20	115.46	110.34
3	B	602	GN1	O3'-C3'-C2'	2.01	113.69	109.65

CONFIDENTIAL VALIDATION REPORT

All (1) chirality outliers are listed below:

Mol	Chain	Res	Type	Atom
3	B	602	GN1	C3'

All (1) torsion outliers are listed below:

Mol	Chain	Res	Type	Atoms
3	B	602	GN1	C1'-O1'-P-OP3

There are no ring outliers.

1 monomer is involved in 2 short contacts:

Mol	Chain	Res	Type	Clashes	Symm-Clashes
3	B	602	GN1	2	0

The following is a two-dimensional graphical depiction of Mogul quality analysis of bond lengths, bond angles, torsion angles, and ring geometry for all instances of the Ligand of Interest. In addition, ligands with molecular weight > 250 and outliers as shown on the validation Tables will also be included. For torsion angles, if less then 5% of the Mogul distribution of torsion angles is within 10 degrees of the torsion angle in question, then that torsion angle is considered an outlier. Any bond that is central to one or more torsion angles identified as an outlier by Mogul will be highlighted in the graph. For rings, the root-mean-square deviation (RMSD) between the ring in question and similar rings identified by Mogul is calculated over all ring torsion angles. If the average RMSD is greater than 60 degrees and the minimal RMSD between the ring in question and any Mogul-identified rings is also greater than 60 degrees, then that ring is considered an outlier. The outliers are highlighted in purple. The color gray indicates Mogul did not find sufficient equivalents in the CSD to analyse the geometry.

CONFIDENTIAL VALIDATION REPORT

Ligand GN1 B 602

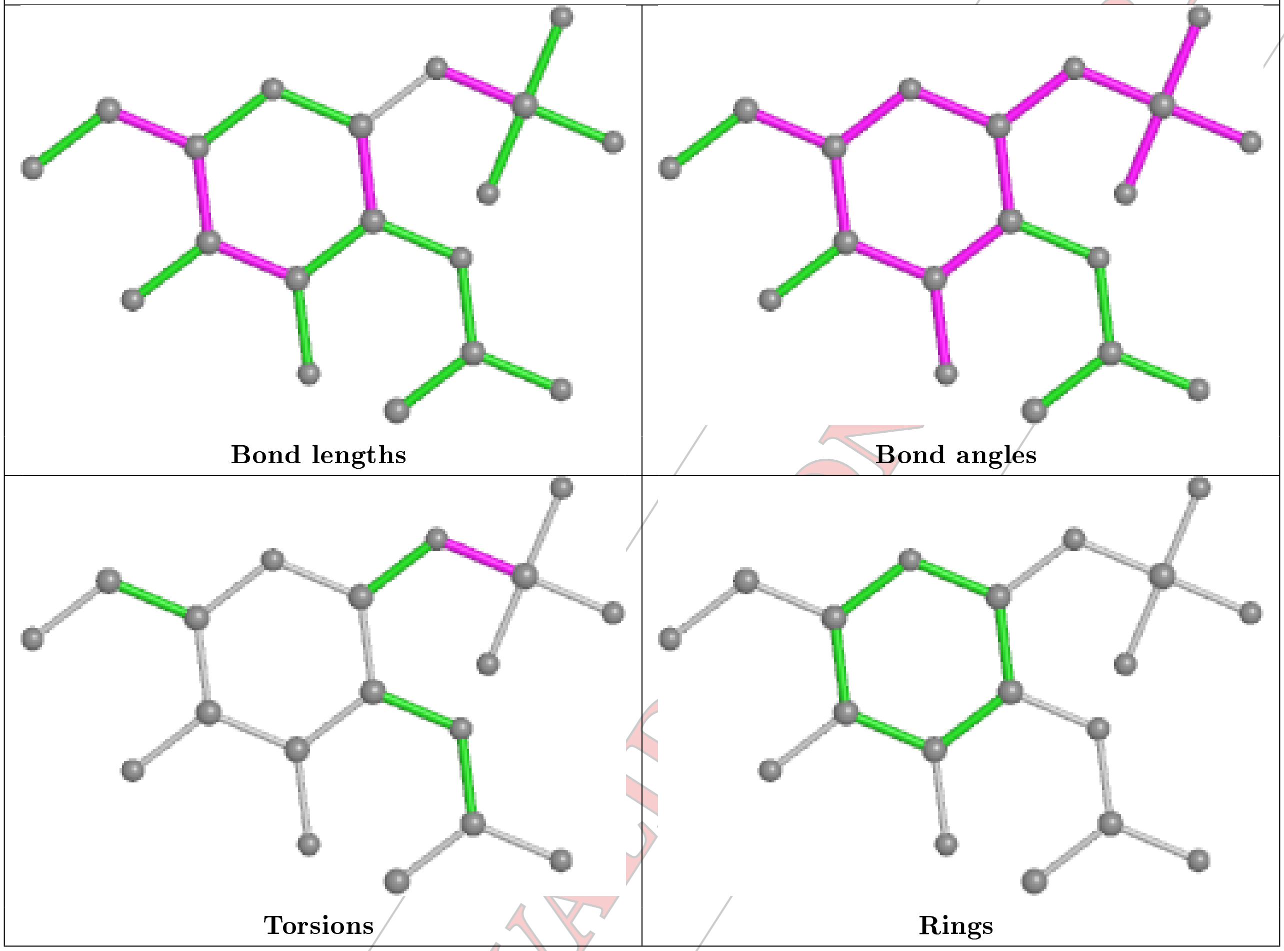

5.7 Other polymers (i)

There are no such residues in this entry.

5.8 Polymer linkage issues (i)

There are no chain breaks in this entry.

CONFIDENTIAL VALIDATION REPORT

6 Fit of model and data (i)

6.1 Protein, DNA and RNA chains (i)

In the following table, the column labelled '#RSRZ> 2' contains the number (and percentage) of RSRZ outliers, followed by percent RSRZ outliers for the chain as percentile scores relative to all X-ray entries and entries of similar resolution. The OWAB column contains the minimum, median, 95th percentile and maximum values of the occupancy-weighted average B-factor per residue. The column labelled 'Q< 0.9' lists the number of (and percentage) of residues with an average occupancy less than 0.9.

Mol	Chain	Analysed	<RSRZ>	#RSRZ>2			OWAB(Å^2)	Q<0.9
1	A	442/509 (86%)	0.18	18 (4%)	37	43	30, 50, 77, 96	0
1	B	463/509 (90%)	0.02	6 (1%)	77	81	28, 43, 68, 97	0
All	All	905/1018 (88%)	0.10	24 (2%)	54	61	28, 46, 73, 97	0

All (24) RSRZ outliers are listed below:

Mol	Chain	Res	Type	RSRZ
1	A	49	ALA	3.9
1	A	240	PHE	3.9
1	A	162	PHE	3.0
1	B	388	ILE	2.9
1	A	227	CYS	2.9
1	B	85	PRO	2.8
1	A	336	GLU	2.8
1	A	179	ILE	2.6
1	B	344	LYS	2.6
1	A	402	ASN	2.6
1	A	137	GLY	2.5
1	B	399	GLU	2.3
1	A	139	GLY	2.3
1	A	141	ARG	2.3
1	A	91	PRO	2.2
1	A	238	SER	2.1
1	A	31	GLU	2.1
1	A	234	ILE	2.1
1	A	442	THR	2.1
1	A	50	PHE	2.1
1	B	30	GLU	2.0
1	A	503	ALA	2.0
1	A	405	LYS	2.0
1	B	470	ILE	2.0

CONFIDENTIAL VALIDATION REPORT

6.2 Non-standard residues in protein, DNA, RNA chains (i)

There are no non-standard protein/DNA/RNA residues in this entry.

6.3 Carbohydrates (i)

There are no carbohydrates in this entry.

6.4 Ligands (i)

In the following table, the Atoms column lists the number of modelled atoms in the group and the number defined in the chemical component dictionary. The B-factors column lists the minimum, median, 95th percentile and maximum values of B factors of atoms in the group. The column labelled 'Q< 0.9' lists the number of atoms with occupancy less than 0.9.

Mol	Type	Chain	Res	Atoms	RSCC	RSR	B-factors(Å^2)	Q<0.9
3	GN1	B	602	19/19	0.95	0.11	30,37,42,46	0
2	PO4	B	601	5/5	0.97	0.11	54,55,58,61	0

The following is a graphical depiction of the model fit to experimental electron density of all instances of the Ligand of Interest. In addition, ligands with molecular weight > 250 and outliers as shown on the geometry validation Tables will also be included. Each fit is shown from different orientation to approximate a three-dimensional view.

CONFIDENTIAL VALIDATION REPORT

Electron density around GN1 B 602:

$2mF_o$-DF_c (at 0.7 rmsd) in gray
mF_o-DF_c (at 3 rmsd) in purple (negative)
and green (positive)

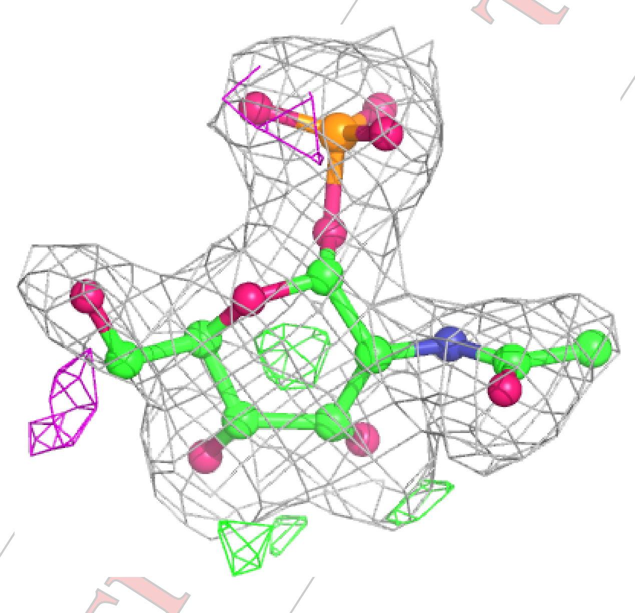

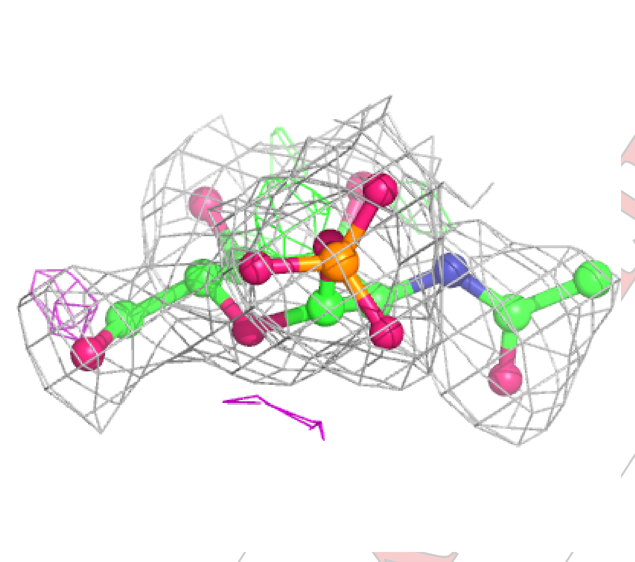

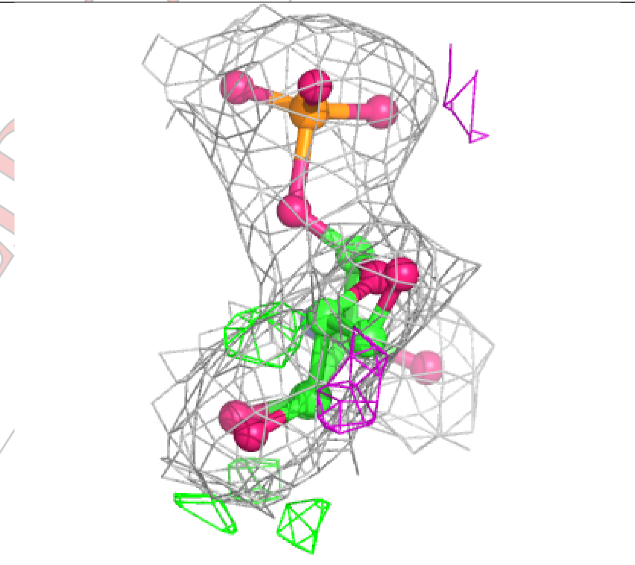

6.5 Other polymers

There are no such residues in this entry.

CONFIDENTIAL
