# Supplementary material for: A mechanism-inspired UDP-N-acetylglucosamine pyrophosphorylase inhibitor
Source: RSC Chem Biol. 2020 Mar 24;1(1):13–25. doi: 10.1039/c9cb00017h (PMC8386105; doi:10.1039/c9cb00017h)
Supplement: CB-001-C9CB00017H-s007 [file CB-001-C9CB00017H-s007.pdf]

Figure S1: **Multiple sequence alignment of UAP1s with *Mt*GlmU**. Multiple sequence alignment of *Af*UAP1, the human AGX1 and AGX2 isoforms, *C. albicans* UAP1 (*Ca*UAP1) and *Mt*GlmU. Secondary structure elements from the *Af*UAP1 structure are shown with α-helices and β-strands in red. The key highly conserved lysine in eukaryotes (Lys437 in *Af*UAP1) is highlighted in cyan and indicated with a green inverted triangle. The insertion loop is underlined and *Af*UAP1 loops are indicated with a line and labelled appropriately as well as helix α*.

Figure S2: **Role of K437 in catalysis. (A) SDS-PAGE of all mutants**. 1. WT, 2. K437A, 3. Y330F, 4. K148M, 5. K437R. All proteins were overexpressed and in the soluble fraction. (B) Activity of K437A, K437R and K437M compared to the wild type. Experiment was done in triplicates and result expressed as mean ± SEM. The Biomol Green assay was performed as described in the Materials and Methods using equal enzyme concentration of 20 nM enzyme, 300 µM UTP and 250 µM GlcNAc-1P terminating after 20 min.

Figure S3: (A). HPLC chromatogram of *me*UTP. The major component (*me*UTP) has a retention time of 6.94. The residual peak of *me*UDP has a retention time of 3.56 min. The major compound comprises 98% of the mixture. (B). *Af*UAP1 activity in the absence of *me*UDP. To investigate the effect of *me*UDP an HPLC assay was performed (Waters Xselect 150 × 4.6 mm column, flow rate 1 mL/ min. Buffer A 100 mM phosphate / 2.5 mM Bu_4HSO_4 in water; Buffer B 100 mM phosphate/ 2.5 mM Bu_4HSO_4 in water-MeCN 1:1). The assay was performed using 5 nM enzyme, 25 µM UTP and 50 µM GlcNAc-1P, for 10 min. The chromatogram shows the production of UDP-GlcNAc (*Rt* = 4.5 min). It also shows a peak corresponding to UDP (*Rt* = 5.55 min) which is thought to be a result of gradual hydrolysis of UTP. The remaining UTP elutes at *Rt* = 9.5 min. (C). Effect of *me*UDP on *Af*UAP1 activity. The experiment was done as described for B in the presence of 500 µM meUDP. The chromatogram shows *me*UDP elutes at *Rt* = 3.56 min; concentration far above the scale. Production of UDP-GlcNAc (*Rt* = 4.5 min) remains unaffected. It also shows a peak corresponding to UDP (*Rt* = 5.55 min) which is taken to be a result of gradual hydrolysis of UTP. The remaining UTP elutes at *Rt* = 9.44 min.
